# LncRNA MALAT1 promotes Erastin-induced ferroptosis in the HBV-infected diffuse large B-cell lymphoma

**DOI:** 10.1038/s41419-024-07209-0

**Published:** 2024-11-12

**Authors:** Xiaofei Bai, Jianguo Li, Xuecong Guo, Yinghui Huang, Xu Xu, Ailing Tan, Yisha Jia, Qiaoyi Sun, Xudong Guo, Jie Chen, Jiuhong Kang

**Affiliations:** 1 Clinical and Translational Research Center of Shanghai First Maternity and Infant Hospital, Shanghai Key Laboratory of Maternal Fetal Medicine, Shanghai Key Laboratory of Signaling and Disease Research, Frontier Science Center for Stem Cell Research, National Stem Cell Translational Resource Center, School of Life Sciences and Technology, Tongji University, Shanghai, 200092 China; 2 Department of Hematology, Changhai Hospital, Naval Medical University, Shanghai, 200433 China

**Keywords:** B-cell lymphoma, Cell death, Long non-coding RNAs

## Abstract

In a retrospective analysis of clinical data from 587 DLBCL (diffuse large B-cell lymphoma) patients in China, 13.8% of cases were associated with HBV (hepatitis B virus) infection, leading to distinct clinical features and poorer prognosis. Moreover, HBV infection has a more pronounced impact on the survival of the GCB (germinal center B-cell-like) type DLBCL patients compared to the ABC (activated B-cell-like) type. In this study, we found that the expression of LncRNA MALAT1 (metastasis-associated lung adenocarcinoma transcript 1) was downregulated in the HBV-infected GCB-type DLBCL patients, and the HBV core protein (HBX) directly inhibited the MALAT1 expression in DLBCL cells. Notably, the overexpression of HBX could attenuate the Erastin-induced ferroptosis in the GCB-type DLBCLs, while MALAT1 re-expression restored sensitivity in the HBX-overexpressing DLBCLs in vitro and in vivo. Mechanistically, MALAT1 competitively hindered SFPQ (splicing factor proline and glutamine-rich) from effectively splicing the pre-mRNA of SLC7A11 (solute carrier family 7 member 11), due to a shared TTGGTCT motif, which impeded the SLC7A11 pre-mRNA maturation and hence diminished its negative regulation on ferroptosis. Together, our study identified HBX’s role in inhibiting MALAT1 expression, promoting SFPQ-mediated splicing of SLC7A11 pre-mRNA, and reducing the GCB-type DLBCL sensitivity to Erastin-induced ferroptosis. Combined with the recent studies that ferroptosis may be involved in the occurrence and development of DLBCL, these findings explain our clinical data analysis that DLBCL patients with low expression of MALAT1 have poorer prognosis and shorter overall survival, and provide a valuable therapeutic target for the HBV-infected GCB-type DLBCL patients.

## Introduction

Diffuse large B-cell lymphoma (DLBCL) is the most prevalent type of non-Hodgkin lymphoma (NHL), accounting for approximately 30–40% of NHL cases [1]. DLBCL is characterized by complex clinical and molecular genetic features, exhibiting rapid progression and high invasiveness. Based on specific B-cell differentiation transcripts, DLBCL is classified into GCB (germinal center B-cell-like) and ABC (activated B-cell-like) subtypes, which possess distinct mutation spectra associated with different pathogenic mechanisms [2]. Despite the CHOP (Cyclophosphamide, Hydroxydaunorubicin, Oncovin, and Prednisone) regimen being the first-line treatment for improving prognosis in most DLBCL patients, 30–40% of patients still face treatment resistance or relapse due to the high heterogeneity of DLBCL [3].

Hepatitis B virus (HBV), which has lymphotropic properties, is closely associated with the occurrence and development of lymphoma. Studies have revealed a significant correlation between the HBV infection status of NHL patients and their progression-free survival (PFS) and overall survival (OS), suggesting that continuous HBV replication may be a key factor affecting chemotherapy outcomes and prognosis [4]. China is a high-incidence area for HBV infection, the retrospective analysis of clinical data from 587 DLBCL patients finds that 13.8% of DLBCL cases are associated with HBV infection, and DLBCL patients with HBV infection exhibit distinct clinical features and worse prognosis [5]. Furthermore, HBV infection has a more pronounced impact on the survival of GCB-type DLBCL patients compared to ABC-type [6]. Therefore, it is imperative to elucidate the molecular mechanisms underlying the poor prognosis of HBV-infected GCB-type DLBCL patients and identify novel therapeutic targets to improve their clinical outcomes.

Ferroptosis, a distinct form of programmed cell death, is characterized by increased membrane oxidative damage, lipid peroxidation, and intracellular reactive oxygen species (ROS) levels [7]. Growing evidence suggests that ferroptosis is closely linked to tumorigenesis, progression, and chemotherapy resistance. Modulating ferroptosis in tumor cells holds promise as an emerging anticancer strategy. The system xc− (cystine/glutamate antiporter) pathway plays a crucial role in regulating ferroptosis by incorporating cystine, then reduced intracellularly to cysteine in a NADPH-dependent manner for glutathione (GSH) synthesis. GPX4, rather than GSH, prevents lipid peroxidation and protects cells from ferroptosis. The ferroptosis inducer Erastin specifically targets the activity of system xc − , leading to decreased intracellular GSH levels and subsequent inactivation of GPX4. Previous studies have demonstrated that DLBCL is more sensitive to Erastin-induced ferroptosis compared to other solid tumor cell lines or hematological tumor cell lines (such as acute myeloid leukemia (AML) and multiple myeloma (MM)) [8]. Additionally, dimethyl fumarate (DMF) exhibits anti-lymphoma activity and can induce ferroptosis in DLBCL cells, with the GCB subtype showing higher sensitivity to DMF-induced ferroptosis compared to the ABC subtype [9]. Our previous studies have also shown that HBV infection significantly reduces the sensitivity of GCB-type DLBCL cells to chemotherapy drugs (cytarabine and methotrexate) [10]. However, the precise association between HBV infection and the incidence of DLBCL, as well as its impact on chemotherapy resistance, particularly in relation to GCB-type DLBCL, remains largely unestablished. Further research is warranted to investigate whether there are key molecules mediating HBV infection and ferroptosis sensitivity in the GCB-type DLBCL cells.

LncRNAs are non-coding RNA molecules longer than 200 nt, participating in various biological processes, including transcription activation, energy metabolism, chromatin modification, and gene expression regulation. Previous studies have revealed the close association between LncRNAs and tumorigenesis, progression, and chemotherapy resistance. Our previous study found that LncRNA NBAT1 (neuroblastoma-associated transcript 1) reduced the sensitivity of HBV-infected DLBCL patients to chemotherapy drugs [10]. Furthermore, LncRNA EIF3J-DT (eukaryotic translation initiation factor 3 subunit J divergent transcript) exhibited high expression in drug-resistant gastric cancer cells and was involved in regulating autophagy and chemotherapy resistance by targeting ATG14 (autophagy-related 14) [11]. It has also been reported that LncRNAs can participate in the regulation of ferroptosis. For instance, LINC00472 promoted ferroptosis and apoptosis in lung cancer cells by interacting with G3BP1 (GTPase activating protein binding protein 1) [12], while LncRNA PMAN (peritoneal metastasis associated long noncoding RNA) suppressed ferroptosis by promoting the cytoplasmic distribution of ELAVL1 (ELAV like RNA binding protein 1) in gastric cancer cells [13]. The mode of action of LncRNAs may vary depending on the cancer types, and no relevant research has been found on the involvement of LncRNAs in regulating ferroptosis in the HBV-infected GCB-type DLBCL cells.

LncRNA MALAT1 has been demonstrated to play a significant role in the occurrence and development of various cancers through the ceRNA regulation network [14]. In our study, we discovered for the first time that MALAT1 was significantly downregulated in the HBX-overexpressing GCB-type DLBCL cells. Knockdown of MALAT1 reduced the sensitivity of GCB-type DLBCL cells to Erastin-induced ferroptosis, similar to the phenotype of HBX overexpression. Mechanistic studies revealed that MALAT1 interacted with the splicing factor SFPQ, inhibiting its splicing activity on the SLC7A11 pre-mRNA, a key gene of the ferroptosis pathway.

## Materials and methods

### Cell culture

The GCB-type DLBCL cell lines SUDHL-4 and DB, and ABC-type DLBCL cell lines TMD-8 and HBL-1, as well as 293FT cells, were obtained from the American Type Culture Collection (ATCC, Manassas, VA, USA). The GCB-type DLBCL and ABC-type DLBCL cell lines were cultured in the RPMI 1640 medium supplemented with 10% FBS (Gibco Life Technologies, 10270-106), while the 293FT cell line was cultured in DMEM medium supplemented with 10% FBS. All cells were maintained at 37 °C with 5% CO_2_ in a humidified atmosphere. The cell lines were authenticated by short tandem repeat (STR) profiling to confirm their identity.

### Tumor xenografts

Six-week-old NOD-SCID male mice (purchased from Charles River) were divided into multiple groups including Ctrl, HBX, HBX + MALAT1, with or without treatment of Erastin (30 mg/kg), IKE (17 mg/kg, Selleck, S8877), and/or Liproxstatin (30 mg/kg, Selleck, S7699). Each group received subcutaneous injections of 1 × 10^7^ cells into the inner side of both hind limbs. The cell suspensions were prepared in a mixture of one part Matrigel matrix gel and two parts serum-free culture medium, with a total volume of 100 μL. Tumor dimensions and body weight were measured every 3 days starting from day 15. Treatments began on day 15 with daily intraperitoneal injections of the appropriate compounds. Tumor volume was calculated using the formula: 0.5 × length × width^2^. Randomization was used to assign animals to different experimental groups, and the investigators were blinded to group allocation during both the experiment and data analysis. Inclusion/exclusion criteria were pre-established, and any animals that did not meet the criteria were excluded from the analysis. All experiments were conducted with the approval of the Institutional Animal Care and Use Committee of Tongji University.

### RNA microarray and sequencing data analysis

The RNA sequencing data and microarray data were downloaded from the TCGA (The Cancer Genome Atlas) and GEO (Gene Expression Omnibus) databases, respectively. The study analyzed independent datasets obtained from GSE10846 and GSE87371, as well as the TCGA-DLBCL datasets.

### Cell proliferation analysis

Cell counting assay and CCK-8 assay were used in detecting cell proliferation. For cell counting assays, cells (8 × 10^4^) were seeded in a 12-well plate and treated with corresponding reagents. Cell numbers were measured at 24, 48, and 72 h using a cell counter. For the CCK-8 assay, cells (3 × 10^3^) were seeded in a 96-well plate and treated with corresponding reagents. The cells were cultured for 24, 48, and 72 h. Then, 10 μL of CCK-8 solution (Beyotime, C0038) was added, and the plate was incubated for 2 h. Absorbance at 450 nm was measured using a SpectraMax iD3 microplate reader.

### Flow cytometry analysis

For cell cycle analysis, the cells were collected and fixed overnight in 1 mL of pre-chilled 70% ethanol according to the instructions of the cell cycle detection kit (Beyotime, C1052). For cell apoptosis detection, the cells were treated under light-protected conditions with propidium iodide (PI) / RNase at 37 °C for 30 minutes according to the instructions of the cell apoptosis detection kit (Beyotime C1062S). Both cell cycle and apoptosis analysis were performed using a FACSCalibur flow cytometer. Data was analyzed using FlowJo software (version 7.6).

### Cell transfection

To perform cell transfection, the synthesized antisense oligonucleotides of MALAT1 (ASO MALAT1) and corresponding negative control were dissolved in the RNase-free water to achieve a final concentration of 20 μM. 1.25 μL of the ASO stock solution was added to 30 μL of 1 × riboFECT™ CP Buffer for dilution. After thorough mixing, add 3 μL of riboFECT™ CP Reagent and incubate at room temperature for 15 minutes. Next, add the transfection mixture dropwise to the cells. Place the cells in a cell culture incubator and incubate for 48 h to assess the efficiency of gene silencing.

### BODIPY staining

Lipid peroxidation was measured using BODIPY 581/591 C11 dye (Invitrogen, D3861). Cells were incubated in a 60 mm dish with 5 μM BODIPY 581/591 C11 dye for 30 minutes at 37 °C. After incubation, the cells were washed with PBS and then immediately analyzed using a flow cytometer (BD FACSVerse).

### Cell death analysis

Cells were stained with PI (Beyotime, C1052) in PBS for 15 minutes to assess cell death. The cells were then subjected to flow cytometry analysis using BD FACSVerse flow cytometer.

### qRT-PCR

The TRIzol reagent (Thermo, Ambion) was used to extract total RNA. The RNA samples were reverse transcribed into cDNA using the cDNA synthesis kit (TaKaRa, Japan). For quantitative analysis, the SYBR Green (Bio-Rad, American) quantitative PCR assay was employed.

### ROS and MDA measurement

The ROS detection Kit (Beyotime, S0033S) was used to measure the ROS level according to product instructions. Briefly, cells were collected and washed twice with PBS, then labeled with 10 µM DCFH-DA, and incubated at 37 °C for 20 minutes. Fluorescence intensity at 488 nm wavelength was detected using a FACSCalibur flow cytometry.

The MDA detection kit (Beyotime, S0131S) was used to measure the MDA level according to product instructions. Cells were collected and lysed, then 200 μL prepared MDA working solution was added and heated at 100 °C for 15 minutes. Centrifuge after cooling, the supernatant was collected, and then measured at 532 nm with a SpectraMax iD3 microplate reader.

### Nuclear run-on assay

Use the Cell Light^TM^ EU Nascent RNA capture kit (RiboBio, C10316) for the detection of nascent RNA. The cells were incubated with a medium containing 0.5 mM 5-ethynyl uridine (RiboBio, China) for 2 h. The TRIzol reagent (Thermo, Ambion) was used to extract the total RNA. Biotin Azide (10 µg RNA, 1 mM Biotin Azide) was added to EU-labeled RNA for 30 minutes. Then, streptavidin-coupled magnetic beads were used to isolate EU-labeled RNA. Gene expression was detected by qRT-PCR.

### RNA stability measurement

Cells were collected and seeded in a 12-well plate, and treated with Actinomycin D (Act D, 5 μg/mL) for 0, 3, 6, and 9 h. RNA was extracted with TRIzol reagent and the RNA samples were reverse transcribed into cDNA using the cDNA synthesis kit (TaKaRa, RR820A). The RNA levels were detected by qRT-PCR.

### RNA immunoprecipitation (RIP)

A total of 1 × 10^6^ cells were collected and lysed with lysate buffer. Protein A and protein G magnetic beads (Bio-Rad, 1614013) were incubated with 4 μg antibodies and rotated at 4 °C for 8 h. After incubation, the magnetic beads were washed five times with a wash buffer. Subsequently, RIP Buffer and cell supernatant were added and incubated at 4 °C overnight. RNA was extracted with TRIzol reagent and reverse transcribed into cDNA. Quantitative analysis of precipitated RNA was performed by qRT-PCR.

### Gene set enrichment analysis (GSEA)

The gene expression profiles used in the GSEA analysis were obtained from the TCGA-DLBCL dataset, and the DLBCL samples were divided into the HBV-positive and HBV-negative groups.

### Western blot

Cells were collected and sonicated in 1 × SDS lysis buffer (Beyotime, China). Perform electrophoresis of an appropriate amount of protein samples in SDS-PAGE gel, and then transfer the samples onto a PVDF membrane. Then, block the membrane with 3% BSA (Amresco, 0332-100 G) for 1 h, followed by overnight incubation with the primary antibody at 4 °C. The primary antibodies used were xCT/SLC7A11 (CST, 12691), GAPDH (Bioworld, AP0063), β-Tubulin (Abclonal, AC008), GPX4 (Proteintech, 67763-1), HBX (Thermo Fisher Scientific, MA1-081), ACSL4 (Proteintech, 22401-1-AP), DHODH (Proteintech, 14877-1-AP), AIFM2 (Santa Cruz Biotechnology, SC-377120), and 4-HNE (Invitrogen, MA5-27570). Then, incubating the membrane with the secondary antibody at room temperature for 1 h. Finally, visualize the bands using the ECL reagent.

### Immunofluorescence

Cells were seeded onto glass coverslips, washed twice with PBS, and the cells were fixed in 4% PFA (Amresco, American) at room temperature for 20 minutes. After fixation, wash the cells twice with PBS for 15 minutes each time. Add 0.3% Triton x-100 permeabilization solution and incubate at room temperature for 30 minutes. Remove the permeabilization solution and add blocking solution, then incubate at room temperature for 1 h. Finally, add the diluted primary antibody and incubate overnight at 4 °C. Remove the primary antibody and incubate with the secondary antibody at room temperature for 1 h. Stain the cell nuclei with the Hoechst33342 staining solution. Capture images using an Olympus microscope.

### Nuclear and cytoplasmic RNA isolation

Collect cells and wash them with pre-chilled PBS, then centrifuge to obtain a cell pellet. Add 200 μL of lysis buffer to resuspend the cell pellet and incubate on ice for 5 minutes. The formulation of the lysis buffer is as follows: Tris pH=8.0 (10 mM), NaCl (140 mM), MgCl_2_ (1.5 mM), and 100% Nonidet P-40 (0.5%). Centrifuge the suspension and collect the supernatant (containing the cytoplasm). Add 1 mL of TRIzol to the supernatant for lysis. Repeat the lysis step twice with the remaining pellet (containing the nuclei) using the lysis buffer. After centrifugation and removal of the supernatant, add 1 mL of TRIzol to precipitate for lysis. Subsequently, proceed with RNA extraction from the respective lysates.

### Statistical analysis

The data were analyzed using GraphPad Prism 8 software and RStudio. Results were presented as the mean ± standard deviation (SD) of three independent replicates. Sample sizes were determined based on power analysis to ensure adequate power to detect a pre-specified effect size. Statistical tests, including t-tests and one-way or two-way ANOVA, were selected based on the specific experimental design and applied appropriately to compare group differences. Prior to analysis, data were assessed to ensure they met the assumptions of the statistical tests, such as normal distribution and homogeneity of variances. Variations within each group were estimated, and comparisons were made only when variances were similar between the groups. A p-value of less than 0.05 was considered statistically significant.

## Results

### HBV infection is associated with ferroptosis and patients’ prognosis

**Fig. 1:**
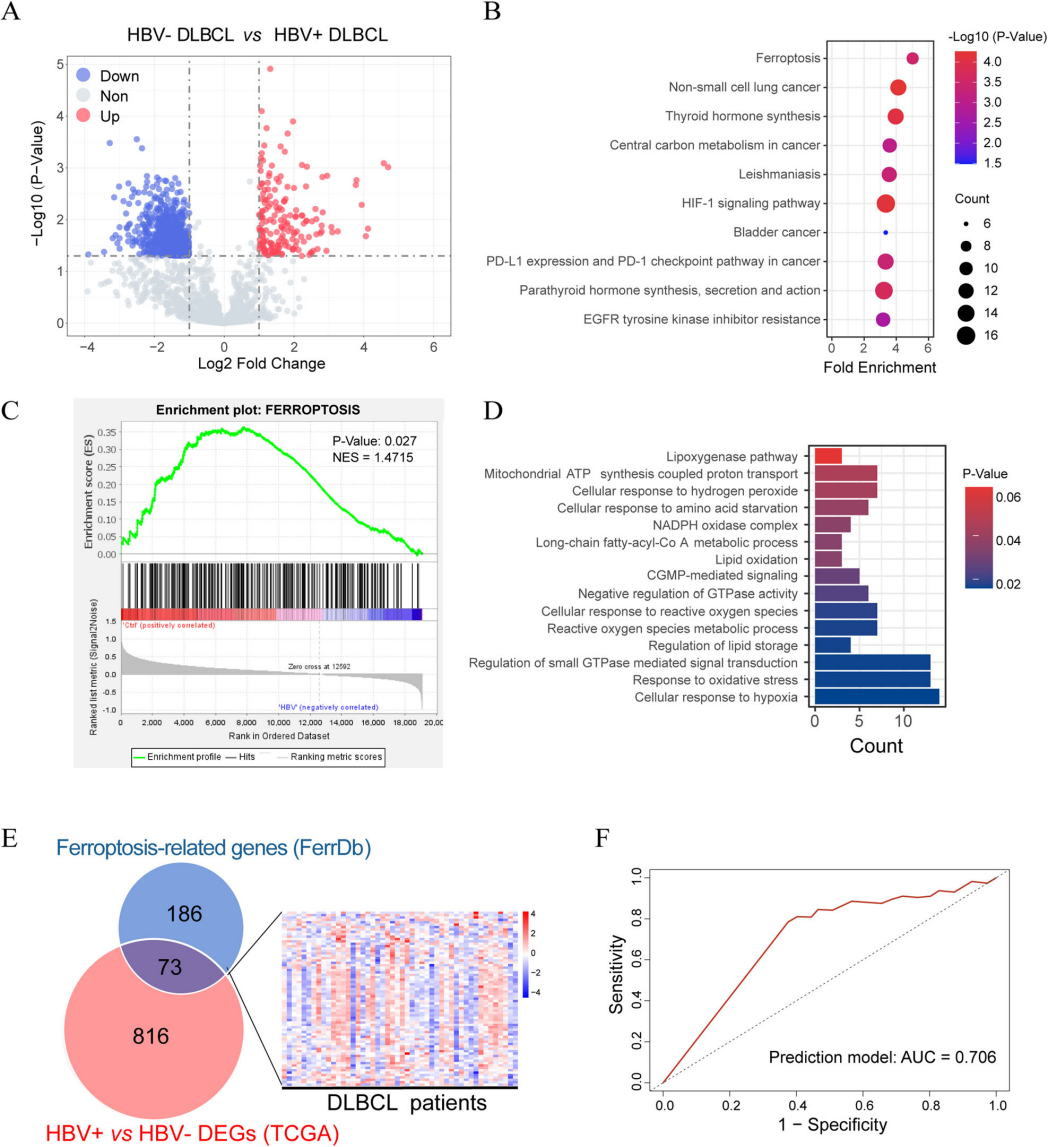
HBV infection is associated with ferroptosis and patients' prognosis. **A** The volcano plot showed 889 differentially expressed genes (DEGs) between the HBV^+^ group and the HBV^-^ group. **B** KEGG enrichment analysis of DEGs in the HBV^+^ and HBV^-^ groups. **C** GSEA analysis revealed the enrichment of DEGs between the HBV^+^ group and the HBV^-^ group in the ferroptosis pathway. **D** Gene ontology (GO) analysis of DEGs in the HBV^+^ and HBV^-^ groups. **E** Venn diagram showed the intersection of DEGs between the HBV^+^ group and the HBV^-^ group and ferroptosis-related genes in the FerrDB database. **F** ROC curve analysis for the prognostic value of DEGs between the HBV^+^ group and the HBV^-^ group of DLBCL patients.

Previous studies have shown that DLBCL patients with HBV infection have a shorter survival time, and HBV infection is considered as an independent factor for the prognosis of DLBCL patients, especially for the GCB-type DLBCL patients [6]. We firstly divided 48 DLBCL patients from the TCGA database (TCGA-DLBCL) into HBV-positive and HBV-negative groups and identified 889 differentially expressed genes (DEGs) (*p* < 0.05 and | Log2 (FC) | > 1.0; upregulated: 179 genes; downregulated: 710 genes) (Fig. 1A). The KEGG pathway analysis revealed that these DEGs were mainly enriched in pathways such as ferroptosis, the HIF-1 signaling pathway, and thyroid hormone synthesis (Fig. 1B). GSEA analysis revealed that individuals with high expression of MALAT1 were enriched in gene signatures positively regulating ferroptosis (Fig. 1C). Furthermore, the enrichment analysis of gene ontology (GO) terms indicated that these DEGs were primarily associated with cellular response to reactive oxygen species, response to oxidative stress, and reactive oxygen species metabolic process (Fig. 1D). In addition, we performed a comparative analysis between the 259 genes related to ferroptosis (FerrDB database) and the 889 DEGs of the HBV-positive group versus HBV-negative group, and 73 genes were found (Fig. 1E). Then, the value of these 73 genes in predicting the prognosis of DLBCL patients was evaluated using ROC curve analysis, yielding an area under the curve of 0.706, which suggested the potential of these genes as prognosis markers of DLBCLs (Fig. 1F). These analyses suggested that HBV infection is significantly associated with ferroptosis and the prognosis of DLBCL patients.

### MALAT1 is significantly downregulated in the HBV-infected GCB-type DLBCLs and closely related to the prognosis of DLBCL patients

**Fig. 2:**
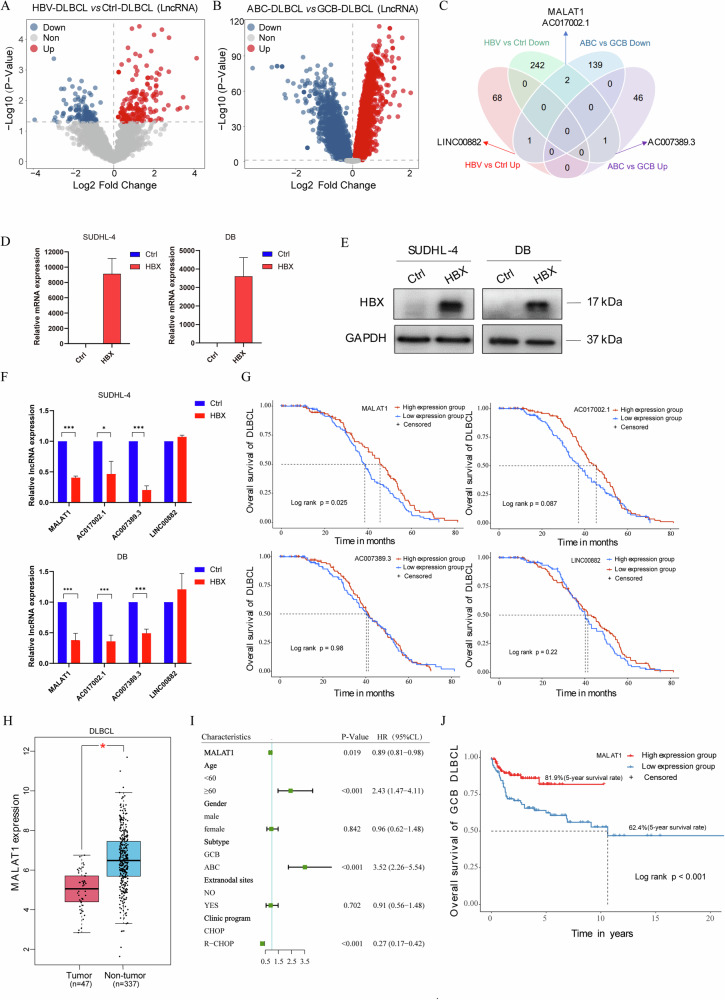
MALAT1 is significantly downregulated in the HBV-infected GCB-type DLBCLs and closely related to the prognosis of DLBCL patients. **A** The volcano plot showed 314 differentially expressed LncRNAs between the HBV group and the Ctrl group. **B** The volcano plot showed 189 differentially expressed LncRNAs between the GCB-type and ABC-type DLBCL patient tissues. **C** Venn diagram showed the candidate LncRNAs associated with poor prognosis in the DLBCL patients with HBV infection. **D** qRT–PCR detection of the mRNA expression level of HBX in the Ctrl and HBX-overexpressing GCB-type DLBCL cells. **E** Western blot detection of the protein level of HBX in the Ctrl and HBX-overexpressing SUDHL-4 and DB cells. **F** qRT–PCR analysis for the expression of MALAT1, AC017002.1, AC007389.3, and LINC00882 in the HBX-overexpressing SUDHL-4 and DB cells compared with control cells. **G** Kaplan-Meier method for analyzing and comparing the survival of individuals with high and low expression of candidate LncRNAs. **H** The expression of MALAT1 in the DLBCL tissues and normal tissues. **I** Univariate regression analysis of clinical pathological variables and MALAT1 expression level in the DLBCL patients. **J** Kaplan-Meier method for analyzing and comparing the survival of individuals with high and low expression of MALAT1. The results were determined in triplicate, and the error bars represented the mean ± SD. * *P* < 0.05 and *** *P* < 0.001.

DLBCLs can be classified into at least two different subtypes according to cellular origin — ABC-type and GCB-type DLBCLs, which have different gene expression profiles and phenotypes [2]. Clinical studies have shown the prognosis of ABC-type DLBCL patients is poorer, but the HBV infection has a worse impact on the prognosis of GCB-type DLBCL patients than ABC-type DLBCL patients [6]. To investigate the role of LncRNAs in the HBV-infected GCB-type DLBCLs, we first analyzed the LncRNA expression profiles of 48 DLBCL patients in TCGA database (TCGA-DLBCL) and identified 314 LncRNAs were associated with HBV infection (|Log2 (FC) | > 1.0 and *P* < 0.05; upregulated: 69 LncRNAs; downregulated: 245 LncRNAs) (Fig. 2A). We also analyzed the DLBCL patient data (GSE10846) and found 142 LncRNAs were highly expressed and 47 LncRNAs were lowly expressed in the GCB-type DLBCL patients compared with ABC-type DLBCL patients (Fig. 2B). Then, we conducted a comparative analysis of the differentially expressed LncRNAs and found that MALAT1, AC017002.1, and AC007389.3 were downregulated in the HBV-infected GCB-type DLBCL patients (Fig. 2C). Since HBX was the major functional fragment of HBV and closely related to the occurrence and development of tumors, we overexpressed HBX in the GCB-type DLBCL cell lines (SUDHL-4 and DB) and evaluated the expression efficiency of HBX using qRT-PCR and western blot (Fig. 2D, E). The expression of MALAT1, AC017002.1, and AC007389.3 was significantly downregulated in the HBX-expressing GCB-type DLBCL cell lines (Fig. 2F). Additionally, MALAT1 and AC007389.3 were highly expressed in the GCB-type DLBCL cell lines (Figure S1). Kaplan-Meier survival analysis of the DLBCL cohort (GSE87371) revealed that only MALAT1 was closely associated with DLBCL patients’ prognosis (Fig. 2G). Further study revealed that MALAT1 was mainly localized in the nucleus of SUDHL-4, DB, HBL-1, and TMD-8 cells by qRT-PCR (Fig. S2). Gene expression profiling interactive analysis (http://gepia.cancer-pku.cn/) also showed that the expression level of MALAT1 was significantly lower in DLBCL tumors (*n* = 47) than in non-tumors (*n* = 377) (Fig. 2H). We further performed the univariate regression analyses of the DLBCL cohort (GSE10846) and found that the expression of MALAT1 served as an independent factor for the prognosis of DLBCL patients (Fig. 2I). Furthermore, the low expression of MALAT1 was also associated with reduced overall survival (OS), according to the GCB-type DLBCL cohorts (GSE10846, Fig. 2J). Collectively, our results indicated that MALAT1, downregulated by HBX, is associated with the poor prognosis of DLBCL patients.

### MALAT1 restores the ferroptosis sensitivity inhibited by HBX in the GCB-type DLBCL cells

**Fig. 3:**
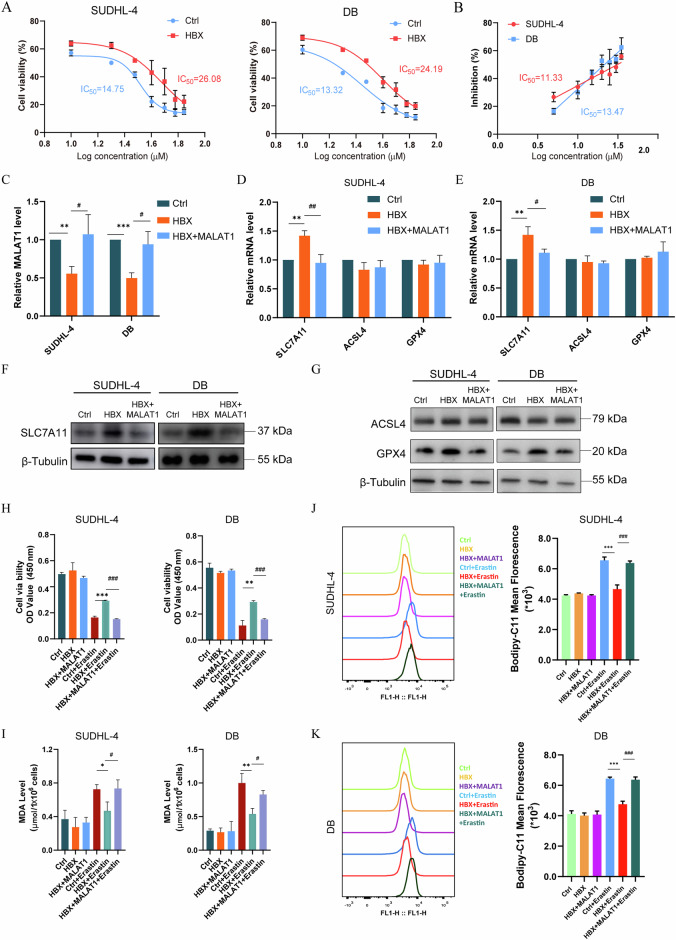
MALAT1 restores the ferroptosis sensitivity inhibited by HBX in the GCB-type DLBCL cells. **A** The CCK-8 assays for cell viability of the Ctrl and HBX-overexpressing SUDHL-4 and DB cells after 24 h of treatment with the indicated doses of Erastin. **B** The CCK-8 detection for the IC_50_ value of Erastin in the Ctrl and HBX-overexpressing SUDHL-4 and DB cells. **C** qRT–PCR analysis for the expression of MALAT1 in the Ctrl, HBX-overexpressing, and HBX-overexpressing cells transfected with MALAT1. “*” represented the significance of HBX versus Ctrl. “#” represented the significance of HBX + MALAT1 versus HBX. **D**–**G**. qRT-PCR analysis (**D** and **E**) and western blot analysis (**F** and **G**) for the expression of SLC7A11, ACSL4, and GPX4 in the Ctrl, HBX-overexpressing, and HBX-overexpressing cells transfected with MALAT1. “*” represented the significance of HBX versus Ctrl. “#” represented the significance of HBX + MALAT1 versus HBX. H-K. The CCK-8 (**H**), MDA **(I**), and lipid peroxidation (**J** and **K**) analysis for the effect of HBX and MALAT1 in SUDHL-4 and DB cells after Erastin treatment. “*” represented the significance of HBX versus Ctrl. “#” represented the significance of HBX + MALAT1 versus HBX. The results were determined in triplicate, and the error bars represented the mean ± SD. ns *P* > 0.05, */# *P* < 0.05, **/## *P* < 0.01, and ***/### *P* < 0.001.

To further investigate the roles of HBX in the ferroptosis of GCB-type DLBCL cells, we used Erastin to induce ferroptosis in SUDHL-4 and DB cell lines. Compared to the Ctrl group, HBX significantly decreased the sensitivity of SUDHL-4 and DB cells to Erastin (Fig. 3A). We determined the IC_50_ values of Erastin in SUDHL-4 and DB cell lines and employed different concentrations of Erastin in the HBX-overexpressing SUDHL-4 (11.33 μM) and DB (13.47 μM) cells (Fig. 3B). Our results showed that HBX significantly inhibited Erastin-induced ferroptosis, but its effect was fully inhibited by the ferroptosis inhibitor Fer-1 (5 μM and 8 μM) (Figure S3). To dissect the effect of MALAT1 on the ferroptosis of DLBCL, we overexpressed MALAT1 in the HBX-expressing SUDHL-4 and DB cells by using lentiviral transfection (Fig. 3C). Next, the expression of SLC7A11, ACSL4 (acyl-CoA synthetase long-chain family member 4), GPX4, AIFM2 (AIF family member 2), and DHODH (dihydroorotate dehydrogenase) were examined by qRT-PCR and western blot. Our results found that MALAT1 significantly restored the increased expression level of SLC7A11 mRNA, but not ACSL4, GPX4, AIFM2, or DHODH (Figs. 3D, E, and S4A). While overexpression of MALAT1 recovered the increased protein levels of SLC7A11 and GPX4 by HBX overexpression, not AIFM2 or DHODH (Figs. 3F, G, and S4B), which was consistent with that SLC7A11 affected the protein level of GPX4, not the GPX4 mRNA level [15] (Figure S5A-D). Moreover, we found that HBX significantly decreased Erastin-induced growth inhibition of SUDHL-4 and DB cells, which were perfectly rescued by MALAT1 re-expression (Fig. 3H). Our results further confirmed that Erastin induced significant cell death in both SUDHL-4 and DB cells. Overexpression of HBX notably reduced the Erastin-induced ferroptotic cell death. Importantly, re-expressing MALAT1 in HBX-overexpressing cells effectively reversed the reduction in cell death caused by HBX (Fig. S6). Additionally, the treatment of ferrostatin effectively inhibited the Erastin-induced cell death in these cells (Fig. S6). We also analyzed the lipid peroxidation (MDA production) and ROS accumulation, which were two important events for triggering ferroptosis, and the elevated levels of MDA production induced by Erastin were markedly repressed by HBX overexpression, while the re-expression of MALAT1 increased the MDA accumulation in both HBX-expressing SUDHL-4 and DB cells (Fig. 3I). Similarly, the ROS generation detected by flow cytometry with the fluorescent probes Bodipy-C11 was repressed by HBX overexpression in Erastin-treated DLBCL cells, which could be significantly reversed by MALAT1 re-expression (Fig. 3J, K). Additionally, we developed an approach to effectively target MALAT1 in vitro relying on the administration of antisense oligonucleotides (ASOs) (Figure S7A). Our results showed that knockdown of MALAT1 significantly increased the mRNA and protein expression levels of SLC7A11 (Fig. S7B and S7C). Consistently, the knockdown of MALAT1 also decreased the ratio of cell death (Figure S7D), reduced the elevation of MDA (Figure S7E), and inhibited the lipid ROS production (Figure S7F and S7G) after Erastin treatment, indicating that MALAT1 regulated the ferroptosis of GCB-type DLBCLs. Besides, our results showed that knockdown of MALAT1 did not affect cell proliferation (Figure S8A-C), cell cycle (Figure S8D), and cell apoptosis (Figure S9) of SUDHL-4 and DB cells. Collectively, overexpression of MALAT1 potentiated the sensitivity of GCB-type DLBCL cells to ferroptosis and reversed the effects of HBX on the Erastin-induced ferroptosis.

### MALAT1 interacts with the splicing factor SFPQ

**Fig. 4:**
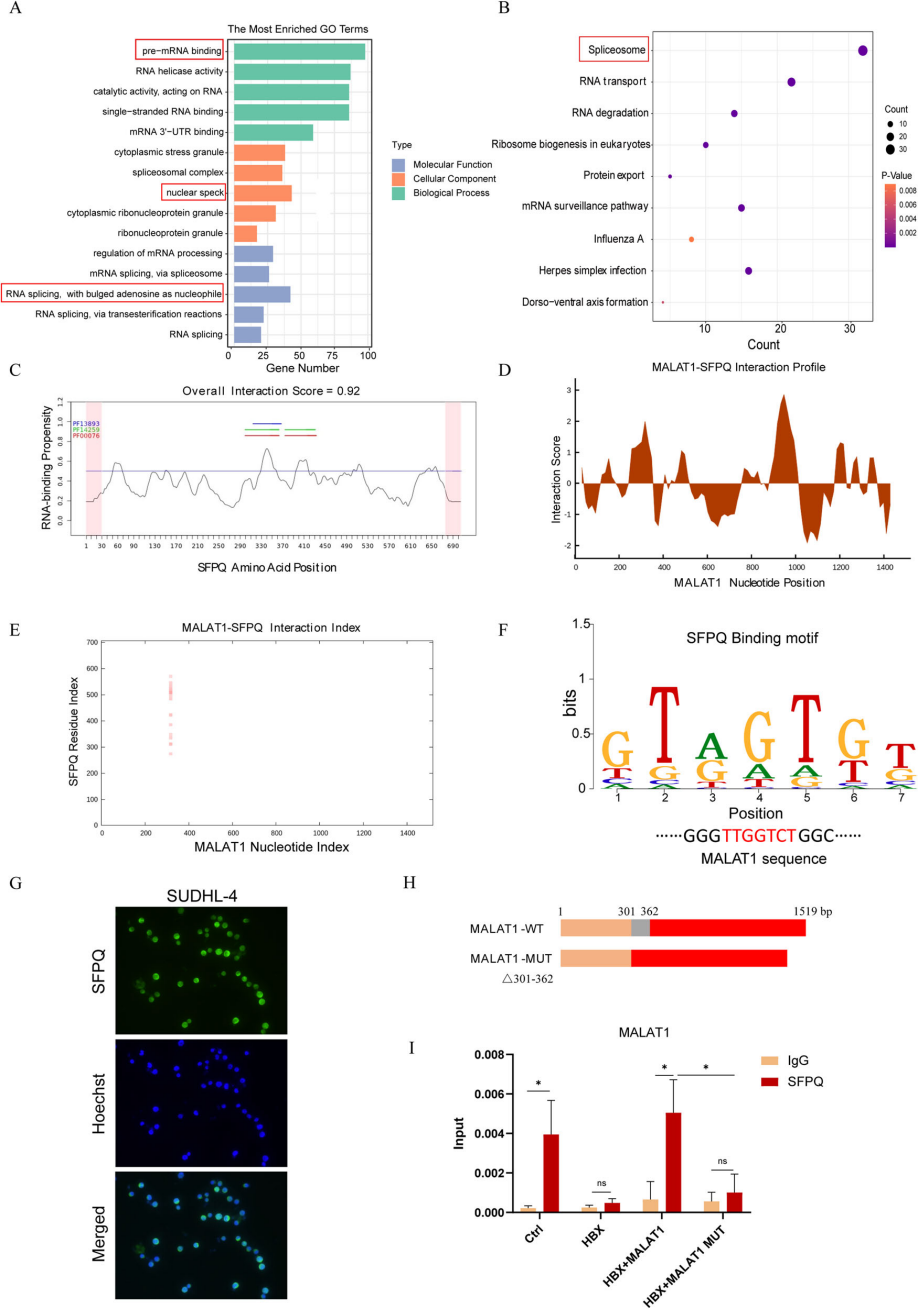
MALAT1 interacts with the splicing factor SFPQ. **A**, **B** GO (**A**) and KEGG enrichment (**B**) analysis of the MALAT1 interaction proteins. **C** The CatRAPID signature module predicted the binding capacity of the SFPQ protein and MALAT1. **D**, **E** The CatRAPID fragments module predicted the interaction spectrum (**D**) and matrix (**E**) between the SFPQ protein and MALAT1. **F** The analysis of the interaction site between MALAT1 and SFPQ. **G** Immunofluorescence staining of the SFPQ (green) in SUDHL-4 cells. Nuclei were stained with Hoechst33342 (blue). Scale bar, 20 μm. **H** Schematic diagram of the full-length MALAT1 and MALAT1 deletion mutant (Δ301–362 nt). **I** RIP assay detected the interaction between SFPQ and wild-type MALAT1 or MALAT1 deletion mutant in SUDHL-4 cells. The results were determined in triplicate, and the error bars represented the mean ± SD. ns *P* > 0.05 and *P < 0.05.

LncRNAs mainly exert their functions and regulate the biological behaviors of cells by binding to DNA, RNA, and proteins. Based on the nuclear localization and structural characteristics of MALAT1, we sought to dissect the mechanistic roles of MALAT1 in ferroptosis by focusing on the interaction between MALAT1 and proteins. We predicted the proteins that potentially bound to MALAT1 by catRAPID omics (Table S1). GO and KEGG analysis (https://david.ncifcrf.gov/) showed that these proteins were mainly localized in the nucleus and enriched in pre-mRNA binding and RNA splicing, associated with the spliceosome formation (Fig. 4A, B). The splicing factor SFPQ (splicing factor proline and glutamine-rich) had the highest possibility of interacting with MALAT1 (Table S2). The catRAPID signature, an algorithm module in the catRAPID server, revealed that an overall score of MALAT1/SFPQ interaction was 0.92 (Fig. 4C). The catRAPID fragments, based on individual interaction propensities of polypeptide and nucleotide sequence fragments, further revealed that the 301–362 nucleotide positions of MALAT1 might bind to 200–600 amino acid residues of SFPQ protein with high propensities (Fig. 4D, E). The binding motif of SFPQ was shown by the cisBP-RNA database (http://cisbp-rna.ccbr.utoronto.ca/) and also found in the MALAT1 sequences (Fig. 4F). Immunofluorescent staining showed that SFPQ was also mainly localized in the nucleus (Fig. 4G). We then employed the MALAT1 deletion mutant to verify the predicted sites of MALAT1/SFPQ interaction (Fig. 4H). The MALAT1-MUT (△301–362) was transfected into HBX-expressing SUDHL-4 cells. As we expected, MALAT1-MUT (△301–362) failed to interact with SFPQ (Fig. 4I), indicating the interaction of wild-type MALAT1 and SFPQ in the GCB-type DLBCL cells. Additionally, we found that there was no significant difference in SFPQ expression between the MALAT1 high-expressed and low-expressed DLBCL patients or HBV-positive and HBV-negative DLBCL patients (Fig. S10A and S10B). Similarly, knockdown of MALAT1 or overexpression of HBX did not affect the mRNA and protein levels of SFPQ (Fig. S10C-F). Therefore, MALAT1 may play an important role in regulating Erastin-induced ferroptosis by interacting with SFPQ.

### MALAT1/SFPQ collaboratively promotes the SLC7A11 intron retention and ferroptosis sensitivity

**Fig. 5:**
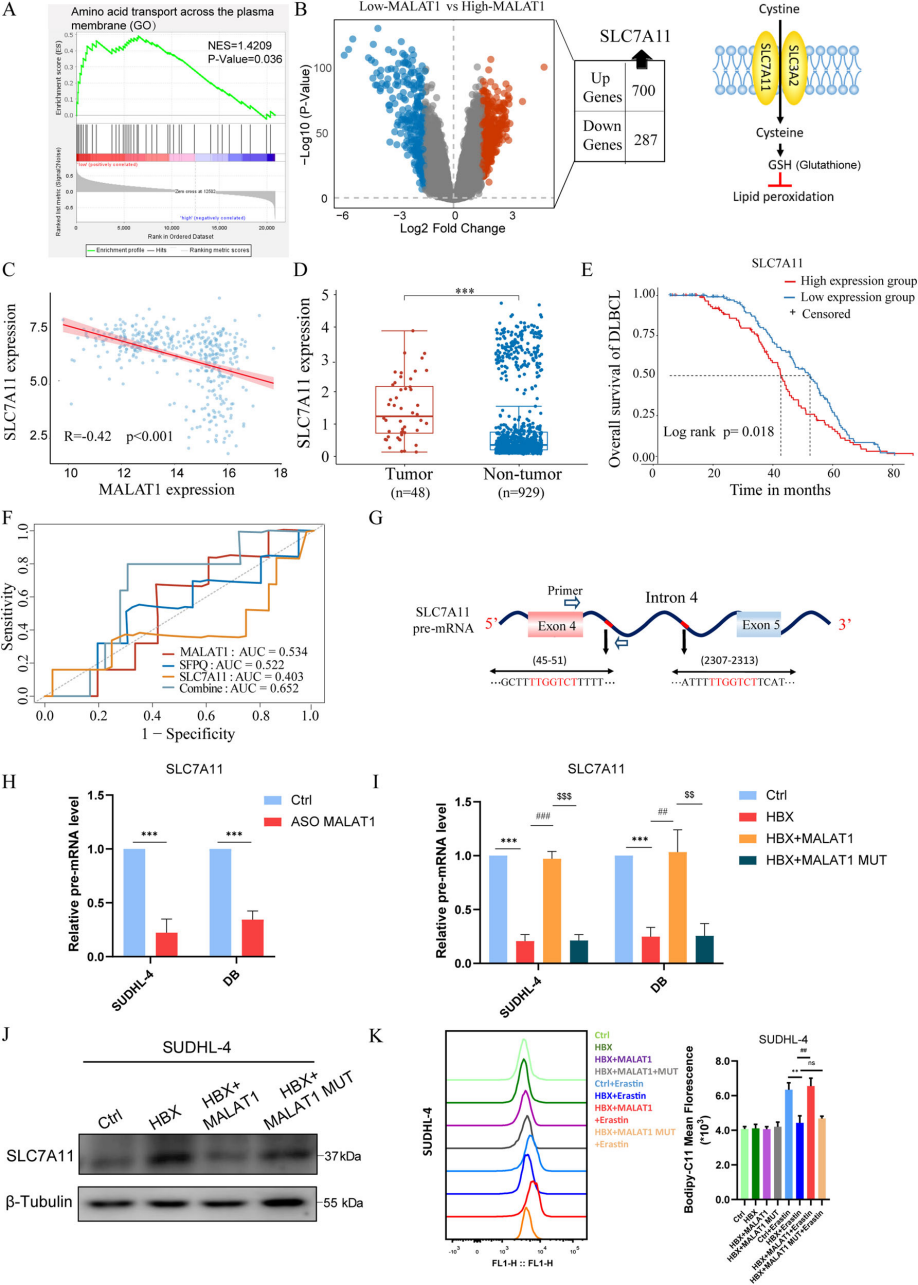
MALAT1/SFPQ collaboratively promotes the SLC7A11 intron retention and ferroptosis sensitivity. **A** The volcano plot showed that 987 genes were differentially expressed between high and low expression of MALAT1 in DLBCL tissue samples. **B** GSEA enrichment analysis of the HBV-regulated genes in DLBCL tissue samples. **C** Pearson correlation analysis between MALAT1 and SLC7A11 expression in the DLBCL patient cohorts (GSE10846). **D** The expression of SLC7A11 in DLBCL tissues and normal tissues. **E** Kaplan-Meier method for analyzing and comparing survival of individuals with high and low expression of SLC7A11. **F** ROC curve analysis for the predictive value of the combination of MALAT1, SFPQ, and SLC7A11 in the prognosis of DLBCL patients. **G** Schematic diagram of the primer design on SLC7A11 pre-mRNA and the intron 4 of SLC7A11 contains two TTGGTCT motifs. **H** The expression level of SLC7A11 transcript containing intron 4 after MALAT1 knockdown. **I** The expression level of SLC7A11 transcript containing intron 4 in the Ctrl, HBX, and HBX+MALAT1 cells. “*” represented the significance of HBX versus Ctrl. “#” represented the significance of HBX+MALAT1 versus HBX. “$” represented the significance of HBX+MALAT1 MUT versus HBX+MALAT1. **J** The SLC7A11 protein level in the Ctrl, HBX, HBX+MALAT1, and HBX+MALAT1 MUT cells. **K** Lipid peroxidation analysis for the effect of HBX, MALAT1, and MALAT1 MUT in SUDHL-4 cells after Erastin treatment. “*” represented the significance of HBX+Erastin versus Ctrl+Erastin. “#” represented the significance of HBX+MALAT1+Erastin versus HBX+Erastin. The results were determined in triplicate, and the error bars represented the mean ± SD. ns *P* > 0.05, ## *P* < 0.01, and ***/### *P* < 0.001.

To explore the mechanisms by which MALAT1 promoted ferroptosis, we examined the mRNA expression profiles in the MALAT1 low- and high-expressed groups from DLBCL tissue samples (GSE10846) (Fig. 5A). We then evaluated the potential ferroptosis-related biological process enriched in HBX-regulated genes by GSEA analysis in the TCGA database of DLBCL. Intriguingly, we found a significant enrichment of the gene signature of amino acid transport across the plasma membrane in the HBX-regulated genes (Fig. 5B). System xc − -mediated cystine uptake for GSH synthesis majorly blocked lipid peroxidation and suppressed ferroptosis [16]. SLC7A11, as the major precursor for glutathione biosynthesis, encoded the system xc− to mediate the uptake of extracellular cystine [17, 18]. According to DLBCL cohorts (GSE10846), we found that the expression of SLC7A11 was negatively correlated with that of MALAT1 (Fig. 5C). The expression level of SLC7A11 in DLBCL tumors (*n* = 48) was also significantly higher than that in non-tumors (*n* = 929) (Fig. 5D). Kaplan-Meier survival analysis of the DLBCL cohort (GSE10846) showed that individuals with high-expressed SLC7A11 had a significantly shorter OS (Fig. 5E). Moreover, the ROC curve regression analysis showed the potential of a combination of MALAT1/SFPQ/SLC7A11 as the prognosis factors of DLBCLs (Fig. 5F). To investigate how MALAT1 functioned on SLC7A11 expression, we first performed the nuclear run-on (NRO) assays to measure the transcriptional activity of the *SLC7A11* gene, while the nascent transcripts of the *SLC7A11* gene were comparable after MALAT1 knockdown (Fig. S11A). Similarly, overexpression of HBX and MALAT1 did not affect the transcriptional activity of the *SLC7A11* gene (Fig. S11B). The quantitative results of mRNA decay rate also revealed that MALAT1 did not affect the SLC7A11 mRNA stability (Fig. S11C and S11D). Then, we speculated that the binding of SFPQ and MALAT1 might affect the alternative splicing of SLC7A11 mRNA. We found that intron 4 of SLC7A11 contained the TTGGTCT motif for SFPQ binding (Fig. 5G). The qRT-PCR data showed that the precursor SLC7A11 mRNA was significantly decreased after MALAT1 knockdown (Fig. 5H). Consistently, re-expression of MALAT1 restored the decreased level of pre-SLC7A11 transcripts, the SLC7A11 protein, and intracellular ROS after HBX overexpression, while the MALAT1-MUT failed (Fig. 5I–K). These results suggested that MALAT1/SFPQ interaction promotes the retention of intron 4 of pre-SLC7A11 transcripts, resulting in the downregulation of SLC7A11 protein. Taken together, MALAT1 might compete with SLC7A11 mRNA to bind SFPQ protein, thereby impairing the SFPQ-mediated SLC7A11 mRNA splicing and potentiating the Erastin-induced lipid ROS production.

### MALAT1 enhances ferroptosis sensitivity in HBX-expressing tumors and is negatively correlated with SLC7A11 in HBV-infected DLBCL patients

**Fig. 6:**
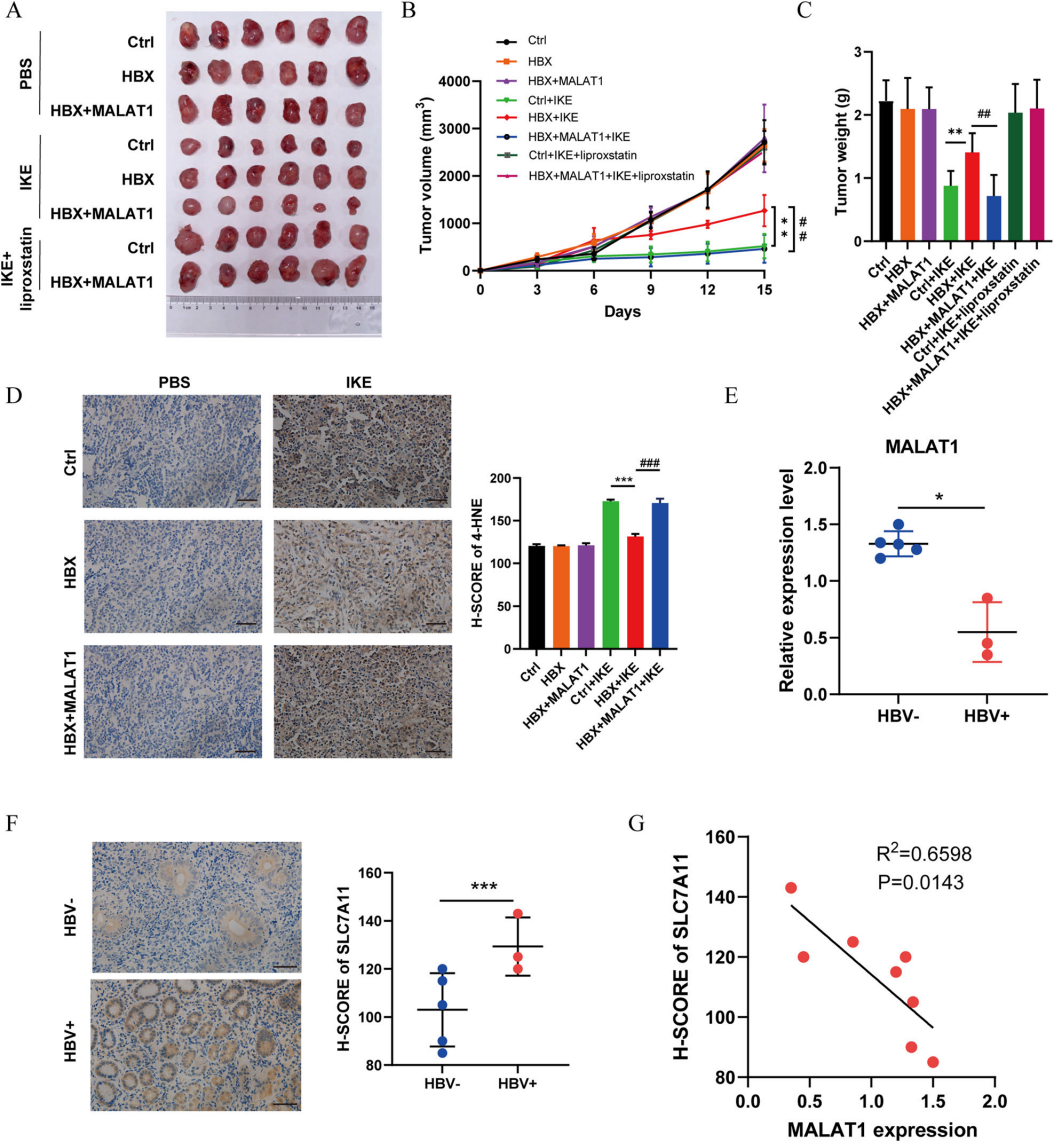
MALAT1 enhances ferroptosis sensitivity in HBX-expressing tumors and is negatively correlated with SLC7A11 in HBV-infected DLBCL patients. **A** Morphological diagrams of tumors in different groups were excised at day 24. **B** The tumor volume of Ctrl, HBX, and HBX+MALAT1 groups was calculated every 3 days with or without IKE/liproxstain treatment. “*” represented the significance of HBX+IKE versus Ctrl+IKE. “#” represented the significance of HBX+MALAT1+IKE versus HBX+IKE. **C** The excised tumor weight of Ctrl, HBX, and HBX+MALAT1 groups at day 24 after IKE/liproxstain treatment. “*” represented the significance of HBX+IKE versus Ctrl+IKE. “#” represented the significance of HBX+MALAT1+IKE versus HBX+IKE. **D** Immunohistochemical detection of 4-HNE in excised tumors. Black scale bar: 100 μm. **E** qRT-PCR analysis for the expression of MALAT1 expression in the GCB-type DLBCL patients with (*n* = 3) or without (*n* = 5) HBV infection. **F** Immunohistochemical detection of SLC7A11 expression in the GCB-type DLBCL patients with (*n* = 3) or without (*n* = 5) HBV infection. Black scale bar: 100 μm. **G** Pearson correlation analysis between MALAT1 and SLC7A11 expression. The results were determined in triplicate, and the error bars represented the mean ± SD. */# *P* < 0.05, **/## *P* < 0.01, and ***/### *P* < 0.001.

To investigate the role of MALAT1 in promoting ferroptosis in vivo, we first conducted experiments using imidazole ketone Erastin (IKE) in mouse xenograft models. We subcutaneously injected control, HBX, and HBX + MALAT1 cells into six-week-old NOD-SCID immunodeficient mice. The IKE treatment significantly reduced tumor volume and weight in the control group, confirming its efficacy in inducing ferroptosis, while the effect of IKE treatment was notably less pronounced in the HBX-expressing group (Fig. 6A–C). Importantly, overexpression of MALAT1 in HBX-expressing tumors restored the sensitivity to IKE treatment (Fig. 6A–C). Moreover, the administration of liproxstatin alongside IKE completely abrogated the tumor-suppressive effect, demonstrating that the anti-tumor effect observed was specifically due to ferroptosis (Fig. 6A–C). Consistent with the results of IKE treatment, HBX overexpression also significantly attenuated the inhibitory effect of Erastin on tumor growth, leading to increased tumor volume and weight, while MALAT1 re-expression effectively rescued these inhibitory effects (Figure S12A-E). Analysis of ferroptosis markers (SLC7A11, ACSL4, and GPX4) and lipid peroxidation levels (4-HNE and MDA) further supported that MALAT1 could counteract the effects of HBX and promote ferroptosis (Fig. 6D and S12F-H). Additionally, our analysis of patient samples revealed that the RNA level of MALAT1 was significantly decreased in the HBV-infected GCB-type DLBCL patients (Fig. 6E), while the SLC7A11 expression was increased (Fig. 6F), with a negative correlation observed between MALAT1 and SLC7A11 expression (Fig. 6G), further supporting the pivotal role of MALAT1 in modulating ferroptosis in the context of HBV-infected DLBCLs.

## Discussion

Epidemiological evidence confirms that HBV infection increases the risk of developing DLBCL [19]. Moreover, the incidence of HBV infection is higher [20], and it may independently contribute to poor prognosis in DLBCL patients [21]. Additionally, the efficacy of chemotherapy is significantly lower in HBV-positive patients compared to HBV-negative patients [22]. These findings highlight the critical role of HBV infection in the occurrence, development, and treatment of B-cell NHL. Ferroptosis is considered a crucial mechanism in suppressing tumor development and eliminating malignant cells, making it a potential approach for cancer treatment. Recent studies have shown that APR-246 can induce ferroptosis in DLBCL cells, regardless of their TP53 mutation status [23]. This suggests that ferroptosis may be involved in the occurrence and development of DLBCL. In our research, we discovered that HBV infection could attenuate Erastin-induced ferroptosis in the GCB-type DLBCL cells. This finding indicated that the inhibition of ferroptosis activity in the HBV-infected GCB-type DLBCL cells may be a significant factor contributing to the poor prognosis of DLBCL patients. Consistent with this, previous studies have found that HBV infection inhibits ferroptosis in liver cancer cells [24], and exosomal miR-222 derived from HBV-infected hepatocytes also inhibits ferroptosis [25]. These findings suggest that ferroptosis inhibition represents a crucial role in HBV infection in its targeted cells. Previous studies have demonstrated that HBV infection of B cell lymphomas can alter B-cell-specific signaling pathways by enhancing overall gene mutations, including CD70 [26, 27], or by increasing non-silent mutations or gene translocations, such as BCL6 [28]. Moreover, HBV viral proteins, especially HBX, directly participate in the regulation of p53 and NF-κB signaling pathways and transcriptional networks in DLBCL [28]. To the best of our knowledge, our study is the first to investigate ferroptosis activity in the HBV-infected GCB-type DLBCL, suggesting that increasing ferroptosis activity may be a potential strategy to improve the poor prognosis of HBV-infected DLBCL patients.

The main mechanisms of ferroptosis involve the catalysis of highly expressed unsaturated fatty acids on the cell membrane by ferrous iron or lipoxygenases, resulting in lipid peroxidation and ultimately inducing cell death [29]. ROS can react with lipids, particularly unsaturated fatty acids, leading to lipid peroxidation and the production of toxic lipid peroxides [30]. Excessive activation of the Ras-MEK pathway may enhance ROS generation by inhibiting Cys2 uptake or VDAC2/3 in mitochondria, thereby sensitizing cancer cells to ferroptosis [31, 32]. Erastin, for instance, blocks the system xc- transporter, effectively reducing intracellular cysteine levels, resulting in ROS accumulation and ferroptosis [8, 33]. Additionally, ferroptosis is characterized by the reduction of the core enzyme GPX4, a key regulator of the antioxidant system [34]. The β-catenin/TCF4 complex, by promoting the expression of GPX4, confers resistance to ferroptosis in gastric cancer cells [34]. Previous studies have shown that exosomal miR-222 derived from HBV-infected hepatocytes aggravates liver fibrosis by inhibiting TFRC-induced ferroptosis [25]. Moreover, HBX directly activates the transcription factor HSF1 to upregulate the expression of HSPA8, inhibiting ferroptosis in liver cancer cells [24]. Combining DMF with FSP1 inhibitors or BH3 mimetics has been shown to synergistically induce ferroptosis in DLBCL cells [9]. In our current research, we found that overexpression of the HBV core functional fragment HBX significantly attenuated the sensitivity of GCB-type DLBCL cells to Erastin-induced ferroptosis by downregulating the expression of MALAT1. This indicated that MALAT1 may serve as a novel target mediating the ferroptosis sensitivity induced by HBV infection in the GCB-type DLBCLs.

MALAT1, a member of the long non-coding RNA family, plays diverse roles in biological processes, including gene regulation, cellular proliferation, and cancer development. Depending on the context and cellular environment, MALAT1 can exhibit both oncogenic and tumor-suppressive functions [35, 36]. It has been reported that MALAT1 regulates the expression of splicing factor SRSF1, promoting the production of splice variants with anti-apoptotic effects [37]. Recently, it is suggested that MALAT1 can regulate ferroptosis through ceRNA mechanisms. By targeting miR-145-5p and regulating MUC1 expression, MALAT1 mediates ferroptosis in EESCs [38]. In our study, we demonstrated that SFPQ was a cooperative molecule of MALAT1 in regulating the ferroptosis of the GCB-type DLBCL cells. SFPQ plays a crucial role in ensuring accurate splicing of introns, and its depletion can lead to increased intron retention, cryptic splicing, premature transcription termination, and polyadenylation [39]. LncRNA NEAT1_2, on the other hand, does not affect the transcription and translation levels of SFPQ, but at least partially mediates cisplatin resistance in liver cancer cells through SFPQ [40]. MALAT1 can bind to SFPQ, resulting in the release of PTBP2 from the SFPQ/PTBP2 complex and promoting the growth and metastasis of colorectal cancer cells [41]. Additionally, as one of the crucial upstream regulatory factors of ferroptosis, SLC7A11 has been extensively documented in recent years to drive ferroptosis resistance [16]. In our study, nuclear MALAT1 bound to SFPQ and inhibited the production of mature SLC7A11 mRNA by blocking the splicing of SFPQ to SLC7A11 pre-mRNA. These discoveries not only elucidated the relationship between HBV infection and ferroptosis in DLBCL cells, but also provided new directions for identifying therapeutic targets in the HBV-infected GCB-type DLBCL patients associated with poor prognosis.

In summary, our research has unveiled a novel function of the MALAT1/SFPQ interaction in mediating ferroptosis by regulating SLC7A11 expression. These findings provide insights into the collaborative regulation of SLC7A11 intron splicing and expression by MALAT1/SFPQ, uncovering a new upstream regulatory mechanism of SLC7A11 in the ferroptosis pathway. Therefore, MALAT1 holds promise as a novel biomarker and offers new insights into ferroptosis-based therapy for the HBV-infected GCB-type DLBCLs.

## Supplementary information

Supplemental information

Original WB images

## Data Availability

The datasets used and/or analyzed during the current study are available from the corresponding author on reasonable request.

## Code availability

The computer code used to generate results central to the conclusions of this paper is available upon request. The code has been developed using R 4.0. Interested researchers can obtain the code by contacting the corresponding author. Please include relevant details regarding the intended use of the code in your request.
