# Supplementary material for: LncRNA MALAT1 promotes Erastin-induced ferroptosis in the HBV-infected diffuse large B-cell lymphoma
Source: Cell Death Dis. 2024 Nov 12;15(11):819. doi: 10.1038/s41419-024-07209-0 (PMC11557927; doi:10.1038/s41419-024-07209-0)
Supplement: Supplementary file 1 — Supplemental information [file 41419_2024_7209_MOESM1_ESM.docx]

**Supplemental Figures**


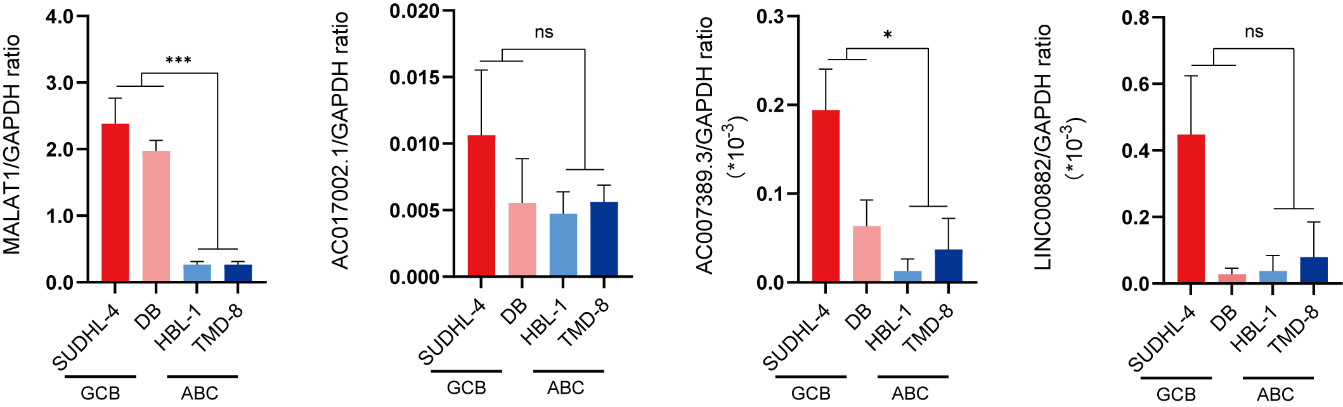


**Figure S1. The expression levels of four candidate LncRNAs in the GCB-type and ABC-type DLBCL cell lines.**

qRT-PCR analysis for the expression of MALAT1, AC017002.1, AC007389.3, and LINC00882 in SUDHL-4, DB, HBL-1, and TMD-8 cells.


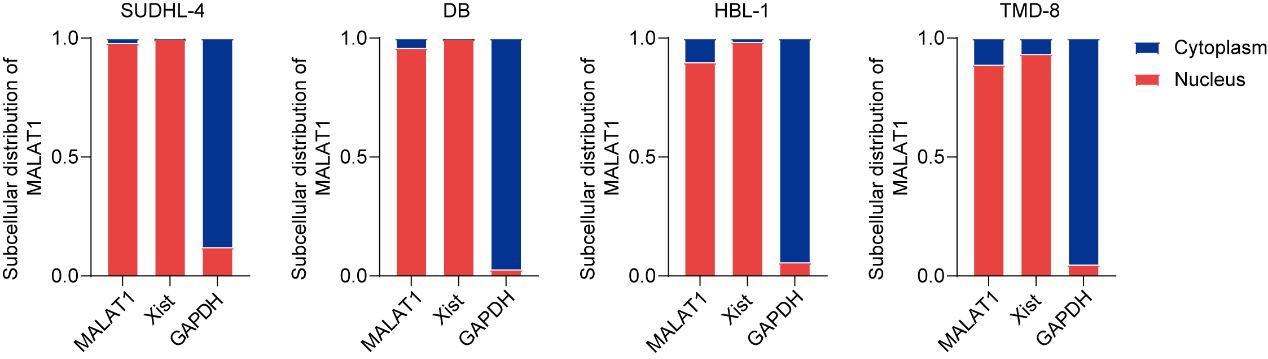


**Figure S2. The subcellular distribution of MALAT1 in DLBCL cells.**

The subcellular distribution of MALAT1 was analyzed by qRT-PCR in SUDHL-4, DB, HBL-1, and TMD-8 cells. GAPDH and Xist genes were used as controls for the cytoplasmic and nuclear fraction, respectively.


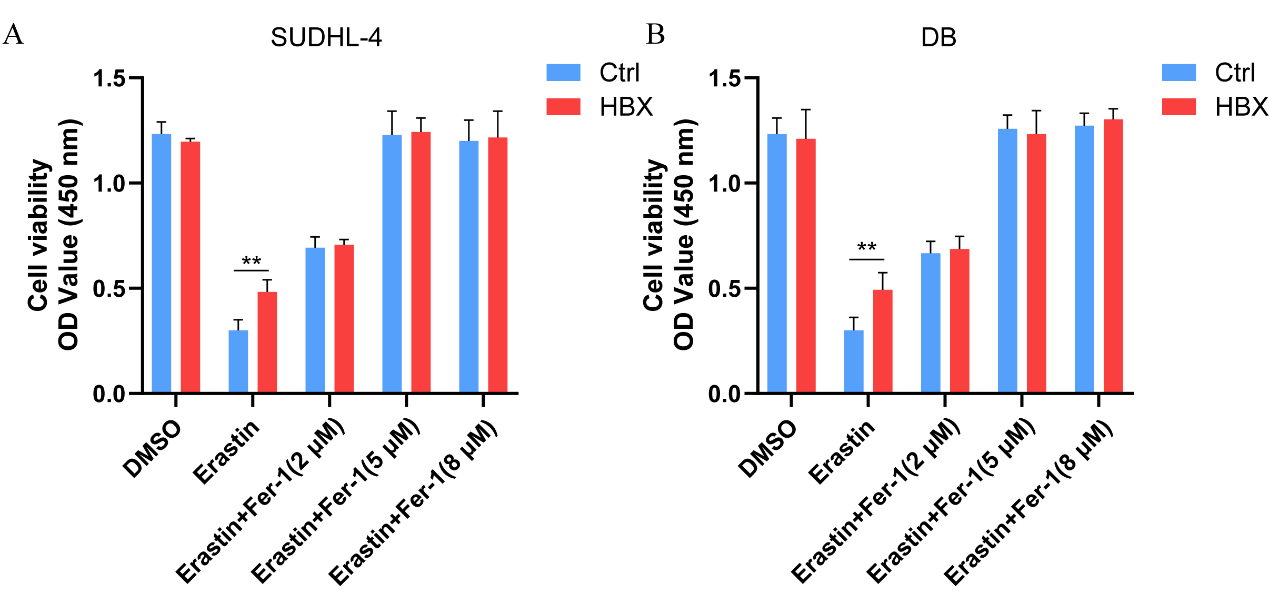


**Figure S3. The treatment of Fer-1 fully reverses the inhibition of ferroptosis caused by HBX overexpression.**

A-B. The CCK-8 assay for cell viability of the Ctrl and HBX-overexpressing SUDHL-4 (A) and DB (B) cells after treatment of DMSO, Erastin (SUDHL-4: 11.33 μM, DB: 13.47 μM), and Erastin+Fer-1 (2 μM, 5 μM, and 8 μM) for 48 h. “*” represents the significance of HBX versus Ctrl.


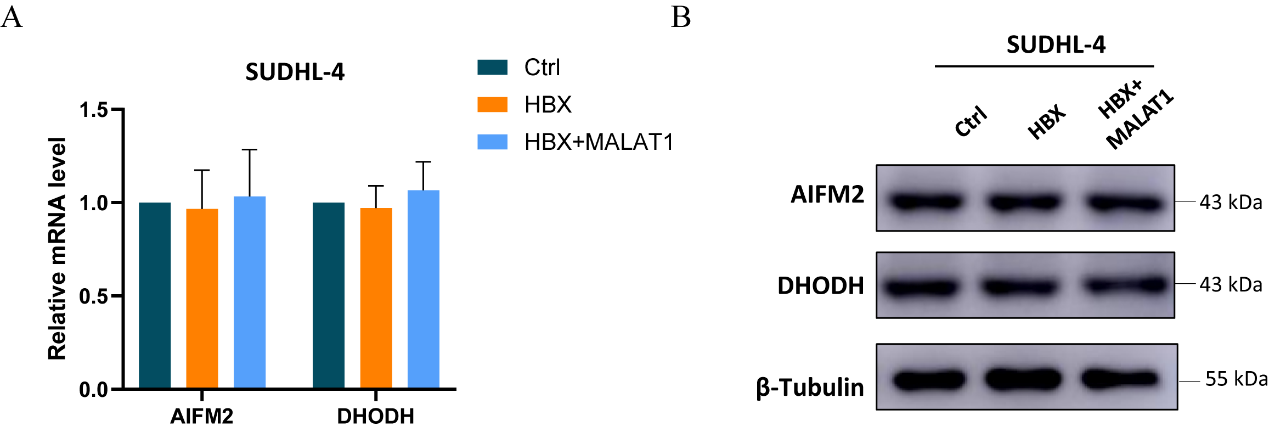


**Figure S4. Overexpression of HBX and MALAT1 has no effect on the mRNA and protein expression levels of AIFM2 and DHODH.**

A-B. qRT-PCR (A) and western blot (B) analysis for the mRNA and protein expression of AIFM2 and DHODH in the Ctrl, HBX, and HBX+MALAT1 groups.


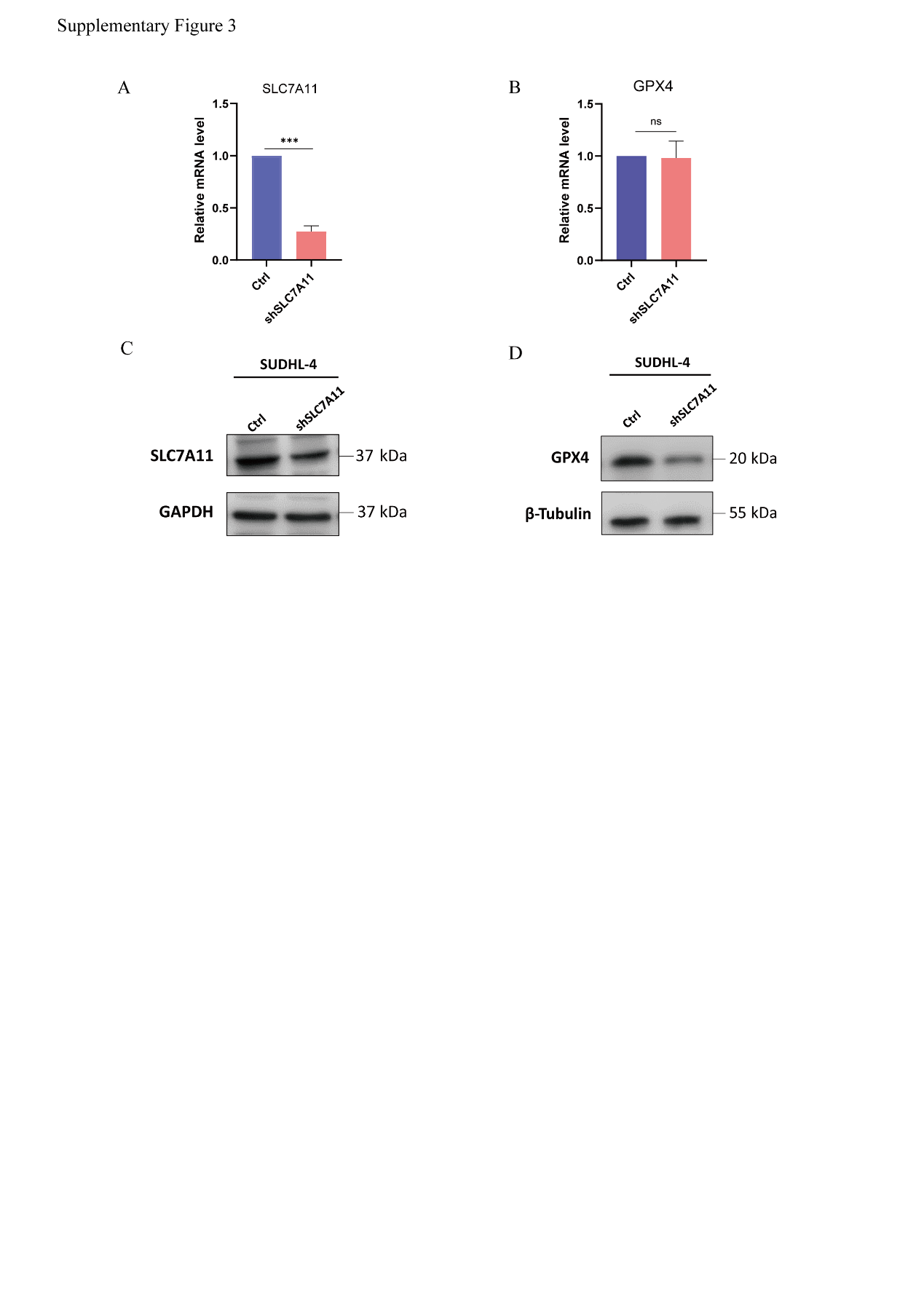


**Figure S5. Knockdown of SLC7A11 significantly reduces the protein level of GPX4.**

A and C. The successful knockdown of SLC7A11 expression was confirmed by qRT-PCR (A) and western blot (C) in SUDHL-4 cells.

B and D. qRT-PCR (B) and western blot (D) analysis for the expression of GPX4 in SUDHL-4 cells after knockdown of SLC7A11.

“*” represents the significance of shSLC7A11 versus Ctrl. The results were determined in triplicate, and the error bars represent as the mean ± SD. ns P > 0.05 and *** P < 0.001.


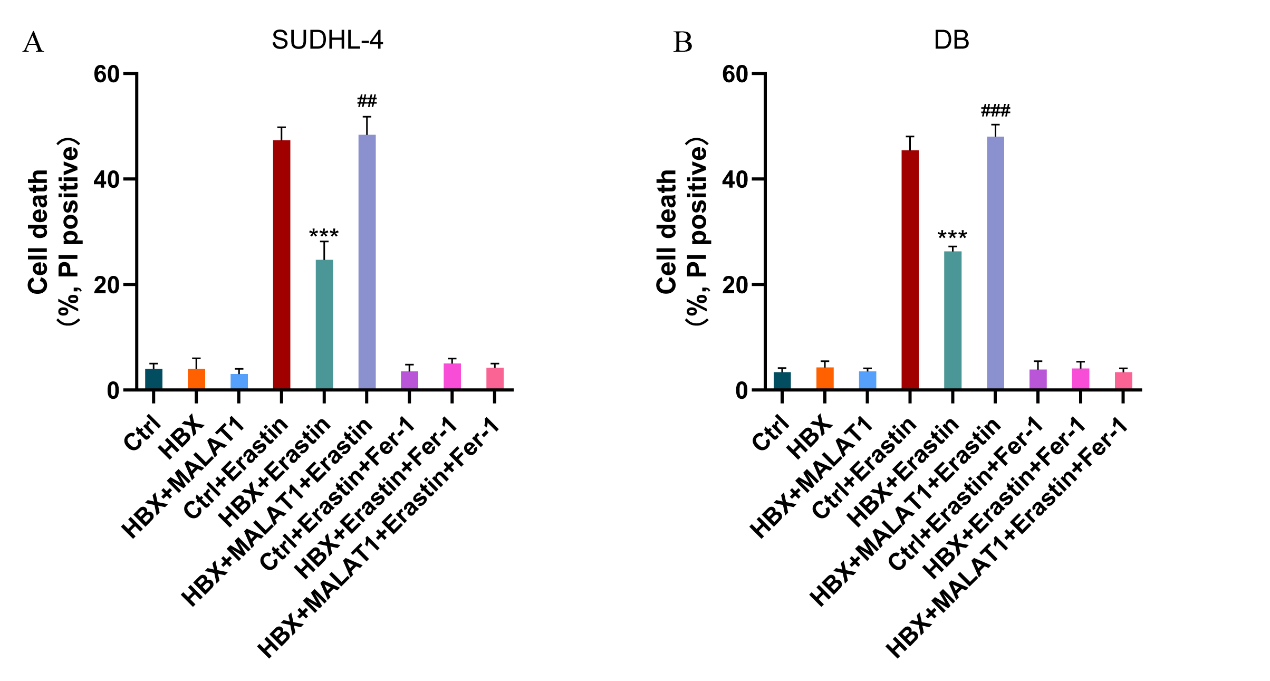


**Figure S6. Overexpression of MALAT1 reverses the HBX-mediated inhibition of Erastin-induced cell death.**

A-B. The cell death analysis of SUDHL-4 (A) and DB (B) cells via PI staining in the Ctrl, HBX, and HBX+MALAT1 groups, along with the treatment of Erastin or Erastin+Fer-1. “*” represents the significance of HBX+Erastin versus Ctrl+Erastin. “#” represents the significance of HBX+MALAT1+Erastin versus HBX+Erastin. The results were determined in triplicate, and the error bars represent as the mean ± SD. ## P < 0.01 and ***/### P < 0.001.


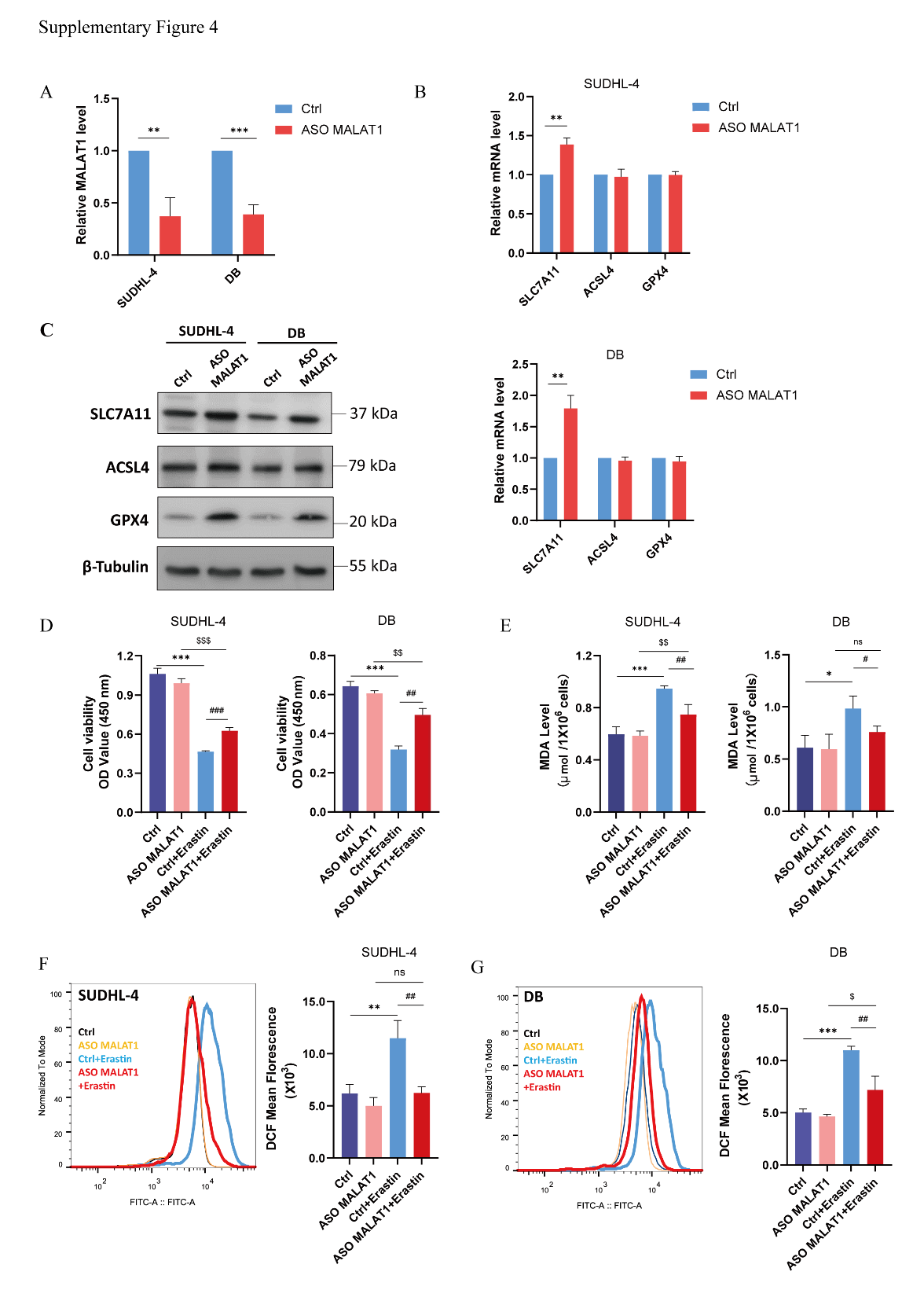


**Figure S7. Knockdown of MALAT1 leads to ferroptosis resistance in the GCB-type DLBCL cells.**

1. The expression level of MALAT1 after ASO treatment in SUDHL-4 and DB cells.

B-C. The expression of SLC7A11, ACSL4, and GPX4 after ASO treatment in SUDHL-4 and DB cells through qRT-PCR (B) and western blot (C) analysis.

D-G. Detecting the effect on cell viability (D), MDA (E), and ROS levels (F and G) of MALAT1 knockdown in SUDHL-4 and DB cells after Erastin treatment. The results were determined in triplicate, and the error bars represent as the mean ± SD. ns P > 0.05, */#/$ P < 0.05, **/## P < 0.01, and ***/### P < 0.001.


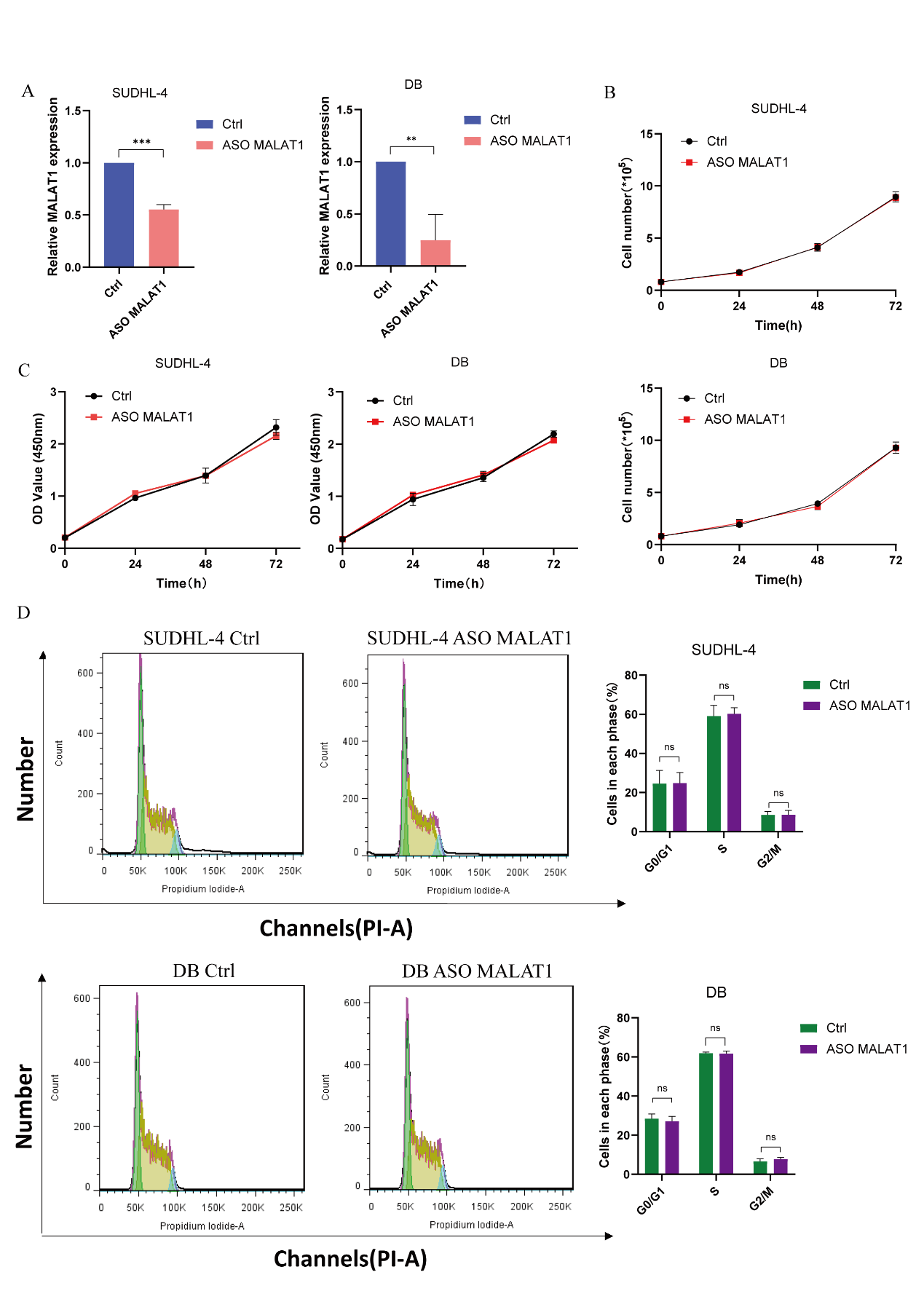


**Figure S8. MALAT1 does not affect the proliferation, cell cycle, and apoptosis of GCB-type DLBCL cells.**

A. The expression level of MALAT1 after ASO treatment in SUDHL-4 and DB cells.

B-C. Cell counting (B) and CCK-8 (C) analysis for SUDHL-4 and DB cell proliferation (24, 48, and 72 h) after MALAT1 knockdown.

D. Flow cytometry analysis for SUDHL-4 and DB cell cycle distribution (24, 48, and 72 h) after MALAT1 knockdown.

“*” represents the significance of ASO MALAT1 versus Ctrl. The results were determined in triplicate, and the error bars represent as the mean ± SD. ns P > 0.05, ** P < 0.01, and *** P < 0.001.


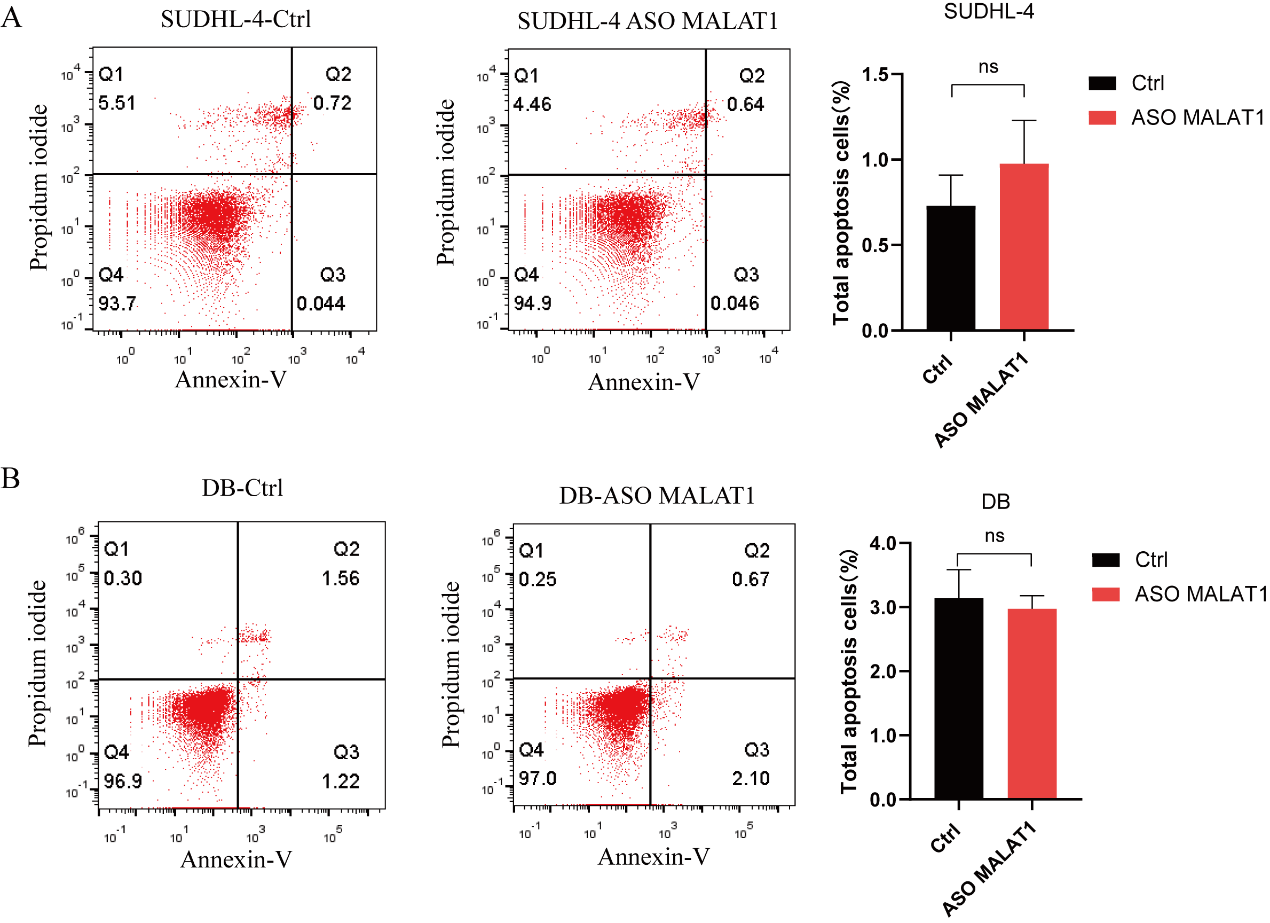


**Figure S9. MALAT1 does not affect the apoptosis of the GCB-type DLBCL cells.**

A-B. Flow cytometry analysis showed for the apoptosis level in SUDHL-4 (A) and DB (B) cells after MALAT1 knockdown. The results were determined in triplicate, and the error bars represent as the mean ± SD. ns P > 0.05.


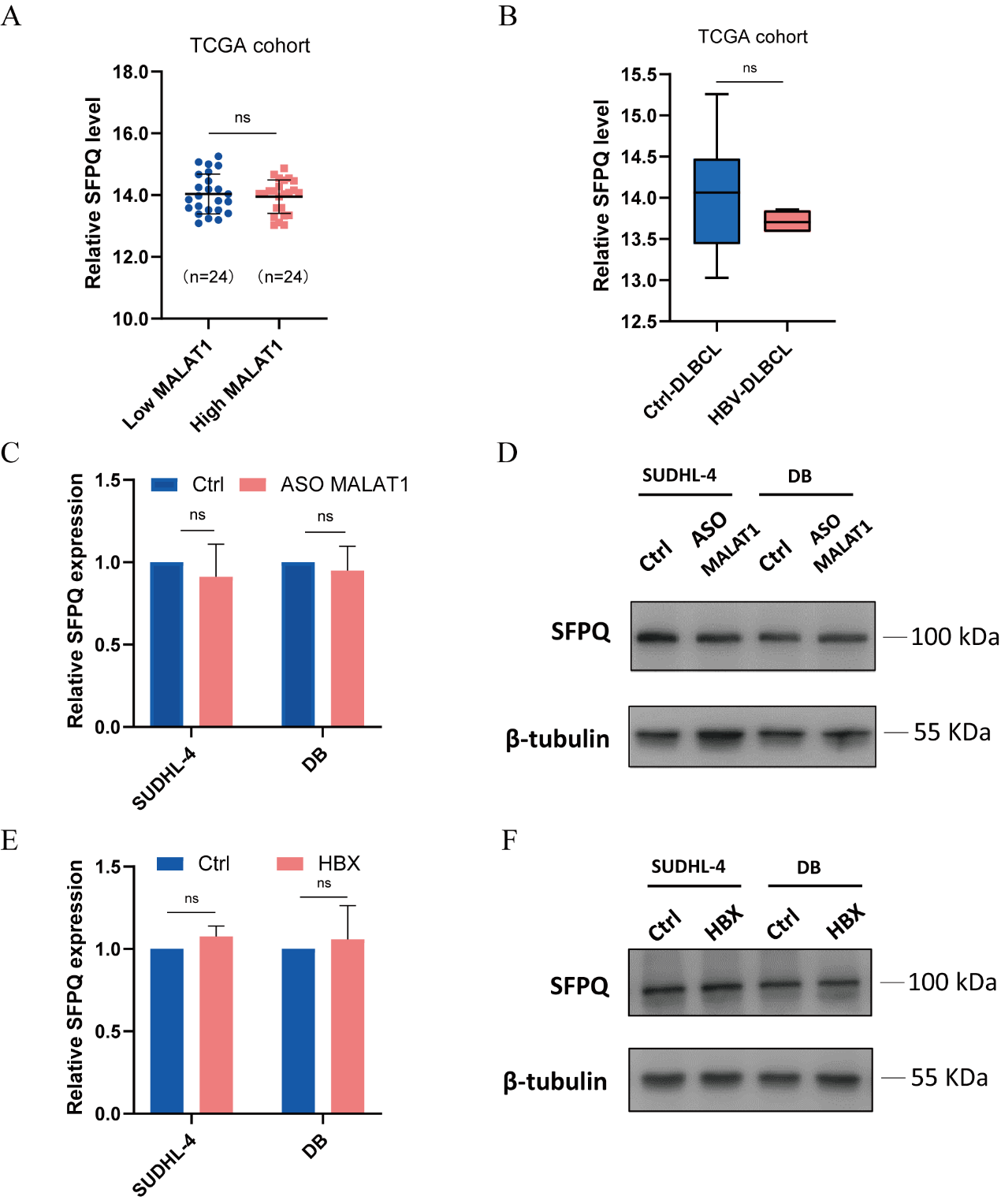


**Figure S10. The expression level of SFPQ is not affected by MALAT1 or HBX expression.**

1. The expression of SFPQ in high and low-MALAT1 DLBCL tissues.
2. The expression of SFPQ in DLBCL tissues with or without HBV infection.

C-D. qRT-PCR (C) and western blot (D) were performed to detect SFPQ expression level after MALAT1 knockdown.

E-F. qRT-PCR (E) and western blot (F) were performed to detect SFPQ expression level after HBX overexpression. The results were determined in triplicate, and the error bars represent as the mean ± SD. ns P >0.05.


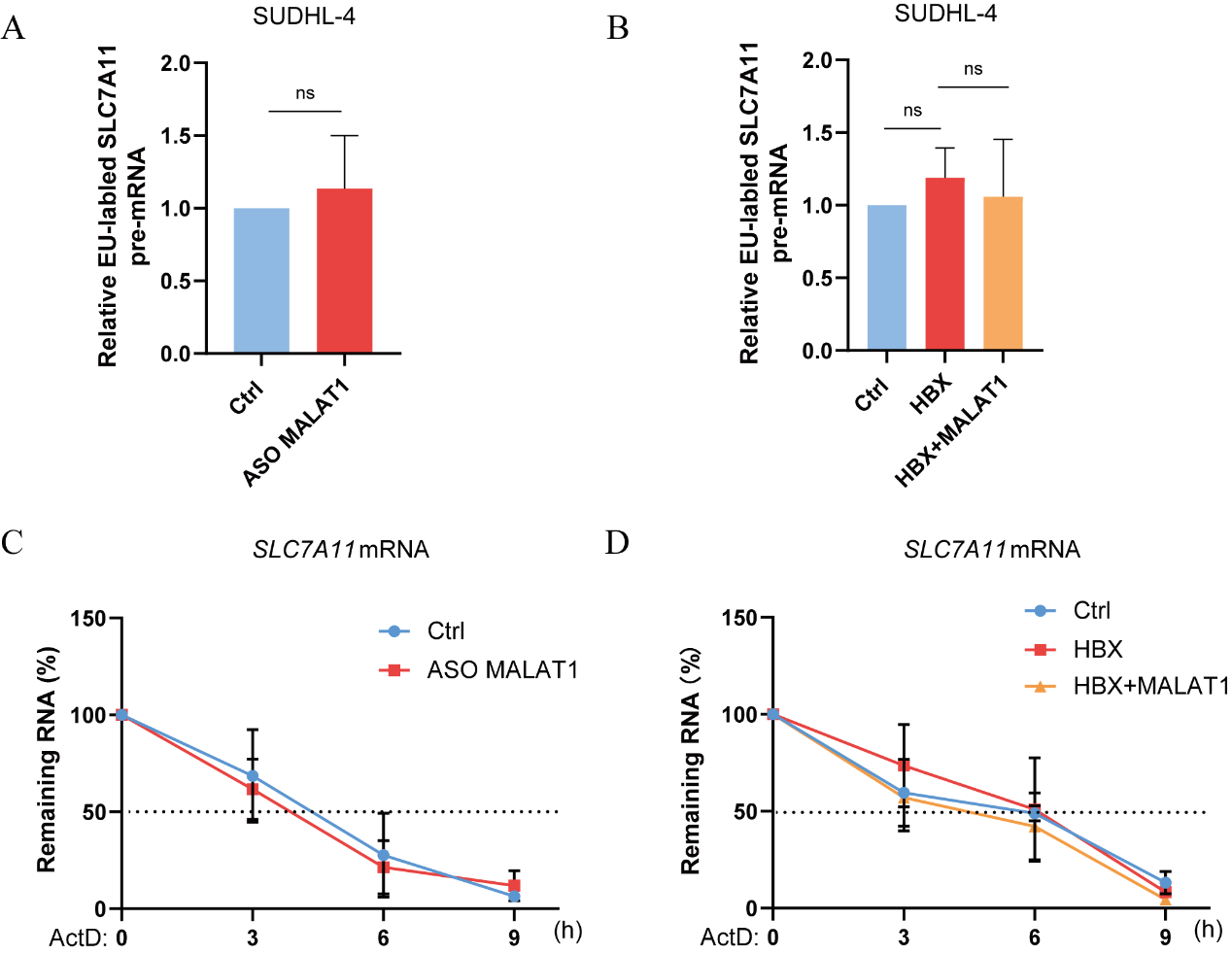


**Figure S11. MALAT1 does not affect the generation of nascent SLC7A11 mRNA and the stability of mature SLC7A11 mRNA.**

1. The nuclear run-on (NRO) assay was performed to determine the transcriptional activity of *SLC7A11* gene after MALAT1 knockdown.
2. The NRO assay was performed to determine the transcriptional activity of *SLC7A11* gene in the Ctrl, HBX, and HBX+MALAT1 groups.
3. qRT-PCR analysis for the effect of MALAT1 on the stability of mature SLC7A11 mRNA after treatment with ActD (4 mg/L) for 0, 3, 6, and 9 h.
4. The stability of mature SLC7A11 mRNA was analyzed in the Ctrl, HBX, and HBX+MALAT1 groups after treatment with ActD (4 mg/L) for 0, 3, 6, and 9 h.


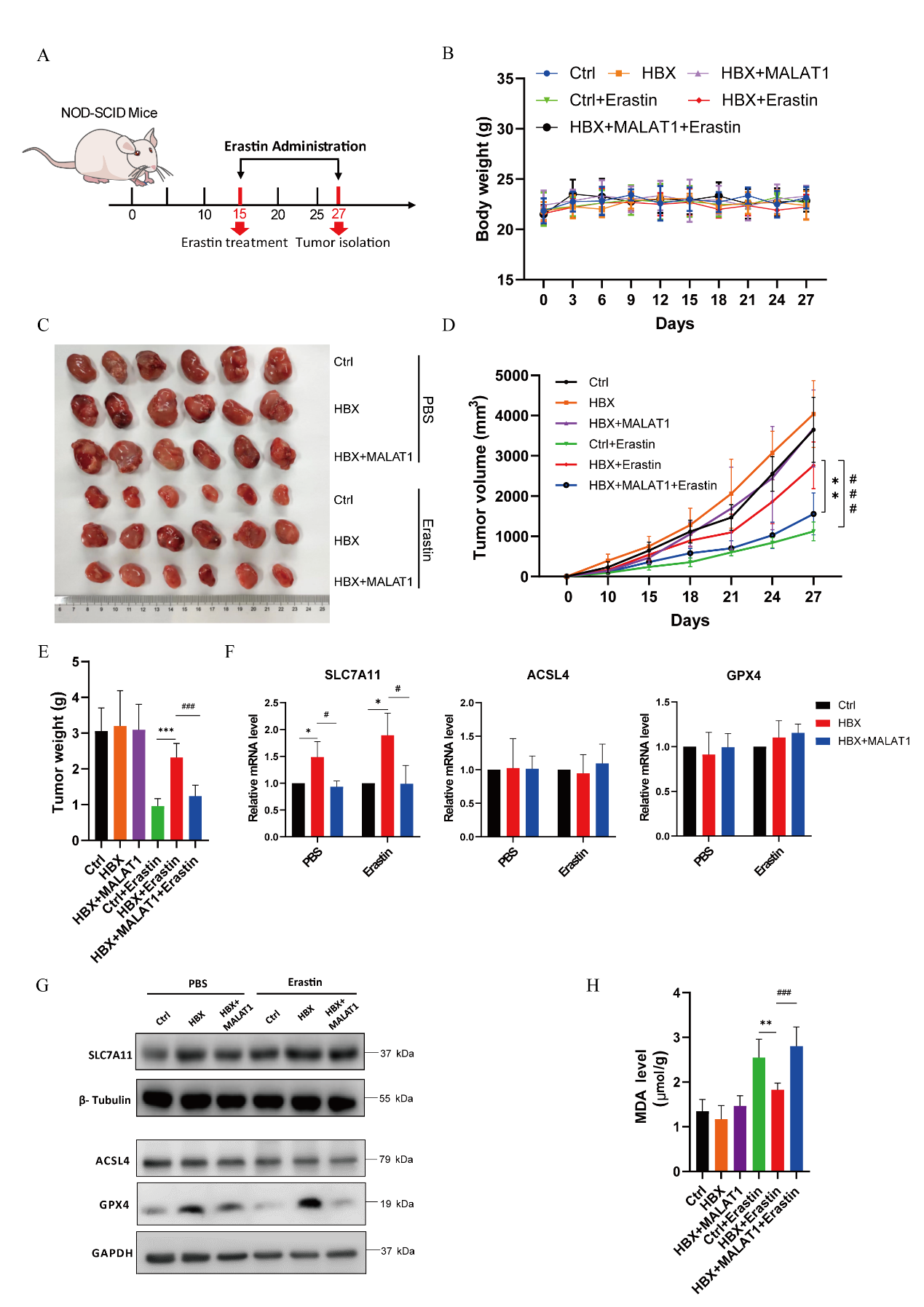


**Figure S12. The overexpression of MALAT1 reverses the HBX-mediated inhibition of Erastin-induced ferroptosis *in vivo*.**

1. Schematic diagram of subcutaneous tumor inoculation and intraperitoneal injection of Erastin.
2. The body weight of Ctrl, HBX, and HBX+MALAT1 groups was calculated every 3 days with PBS or Erastin treatment.
3. Morphological diagrams of tumors in different groups were excised at day 27.
4. The tumor volume of Ctrl, HBX, and HBX+MALAT1 groups was calculated every 3 days with or without Erastin treatment.
5. The excised tumor weight of Ctrl, HBX, and HBX+MALAT1 groups at day 27 after PBS or Erastin treatment.
6. qRT-PCR analysis for the expression of SLC7A11, ACSL4, and GPX4 in xenograft tumors.
7. Western blot analysis for the expression of SLC7A11, ACSL4, and GPX4 in xenograft tumors.
8. The MDA level was detected in excised tumors at day 27.

“*” represents the significance of HBX+Erastin versus Ctrl+Erastin. “#” represents the significance of HBX+MALAT1+Erastin versus HBX+Erastin. The results were determined in triplicate, and the error bars represent the mean ± SD. ns P > 0.05, */# P < 0.05, **/## P < 0.01, and ***/### P < 0.001.

| **Table S1. The potential proteins that interact with MALAT1.** | | | | |
| --- | --- | --- | --- | --- |
| SwissProt ID | Protein | Z-score | Domain | Star Rating Score |
| P23246 | SFPQ_HUMAN | 0.37 | Yes | 2.84 |
| Q13242 | SRSF9_HUMAN | 0.17 | Yes | 2.76 |
| Q9UNW9 | NOVA2_HUMAN | 0.14 | Yes | 2.7 |
| Q15366 | PCBP2_HUMAN | 0.14 | Yes | 2.62 |
| Q01130 | SRSF2_HUMAN | 0.14 | Yes | 2.58 |
| Q15717 | ELAV1_HUMAN | 0.1 | Yes | 2.38 |
| Q15365 | PCBP1_HUMAN | 0.1 | Yes | 2.36 |
| O75525 | KHDR3_HUMAN | 0.1 | Yes | 2.29 |

| **Table S2. The MALAT1 / SFPQ interaction regions.** | | | |
| --- | --- | --- | --- |
| SFPQ | MALAT1 | Score | dp |
| 501-552 | 301-362 | 10.01 | 0.32 |
| 507-558 | 301-362 | 9.23 | 0.28 |
| 301-352 | 301-362 | 9.16 | 0.28 |
| 407-458 | 301-362 | 8.93 | 0.26 |
| 526-577 | 301-362 | 8.63 | 0.26 |
| 476-527 | 301-362 | 8.54 | 0.26 |
| 376-427 | 301-362 | 8.43 | 0.26 |
| 326-377 | 301-362 | 8.42 | 0.26 |

| **Table S3. The primers for screening, validation, and vector construction.** | | |
| --- | --- | --- |
| HBX | Forward | TTCTCCGCCTGTTCTACCGA |
|  | Reverse | TGAAAGTCCAAGAGTCCTCTTATGC |
| MALAT1 | Forward | ACTTGTTCCTGTGGGCTTCA |
|  | Reverse | AGGCACTGATCACTTTAGAGGC |
| AC017002.1 | Forward | TCCTGCTTCTTCGTCACAGG |
|  | Reverse | ATCTGGACACGCACTTCACC |
| AC017053.1 | Forward | GATCCAGGCCACTGTAACATCA |
|  | Reverse | TTCAGGCGTTGATTGCAGC |
| LINC00882 | Forward | GCCGATACTTGACCTACGCA |
|  | Reverse | AGATGGCAGGTGCAATCACA |
| SLC7A11 | Forward | GCGTGGGCATGTCTCTGAC |
|  | Reverse | GCTGGTAATGGACCAAAGACTTC |
| ACSL4 | Forward | CATCCCTGGAGCAGATACTCT |
|  | Reverse | TCACTTAGGATTTCCCTGGTCC |
| GPX4 | Forward | GAGGCAAGACCGAAGTAAACTAC |
|  | Reverse | CCGAACTGGTTACACGGGAA |
| SFPQ | Forward | CTTCGAGTTCGCTTTGCCAC |
|  | Reverse | GACTGGACGAGGAGTTGTCG |
| AIFM2 | Forward | AGACAGGGTTCGCCAAAAAGA |
|  | Reverse | CAGGTCTATCCCCACTACTAGC |
| DHODH | Forward | CCACGGGAGATGAGCGTTTC |
|  | Reverse | CAGGGAGGTGAAGCGAACA |
| SLC7A11 pre-mRNA | Forward | CTGTAGTGATGGTCCTAAATAGC |
|  | Reverse | TGCTGTTCCCGTATTGACTCCTC |
| Xist | Forward | GCATAACTCGGCTTAGGGCT |
|  | Reverse | TCCTCTGCCTGACCTGCTAT |
| GAPDH | Forward | GGAGCGAGATCCCTCCAAAAT |
|  | Reverse | GGCTGTTGTCATACTTCTCATGG |
| shSLC7A11 | Forward | CCGGGAGTCTGGGTGGAACTCCTCATAATCTCGAGATTATGAGGA  GTTCCACCCAGACTCTTTTTG |
|  | Reverse | AATTCAAAAAGAGTCTGGGTGGAACTCCTCATAATCTCGAGATTAT  GAGGAGTTCCACCCAGACTC |
| OE-MALAT1 | Forward | GCGCACCGGTTTTCCAAAGTTTGCATGTTAACTTT |
|  | Reverse | GCGCGAATTCCTTCGCATACGTGTGTCTGCTGAGT |
| OE-MALAT1-MUT1 | Forward | GCGCACCGGTGGCAAATATTGGCAATTAGTTGGCA |
|  | Reverse | GCGCGAATTCGAAGGAAAACAGATACAGTATCAAA |
| OE-MALAT1-MUT2 | Forward | GCGCACCGGTTGACATTAACTACAATTATGGGAAA |
|  | Reverse | GCGCGAATTCTTTATTATTTTGAATGATTTAATGG |
