# Supplementary material for: LncRNA MALAT1 promotes Erastin-induced ferroptosis in the HBV-infected diffuse large B-cell lymphoma
Source: Cell Death Dis. 2024 Nov 12;15(11):819. doi: 10.1038/s41419-024-07209-0 (PMC11557927; doi:10.1038/s41419-024-07209-0)
Supplement: Supplementary file 2 — Original WB images [file 41419_2024_7209_MOESM2_ESM.pdf]

Figure2E

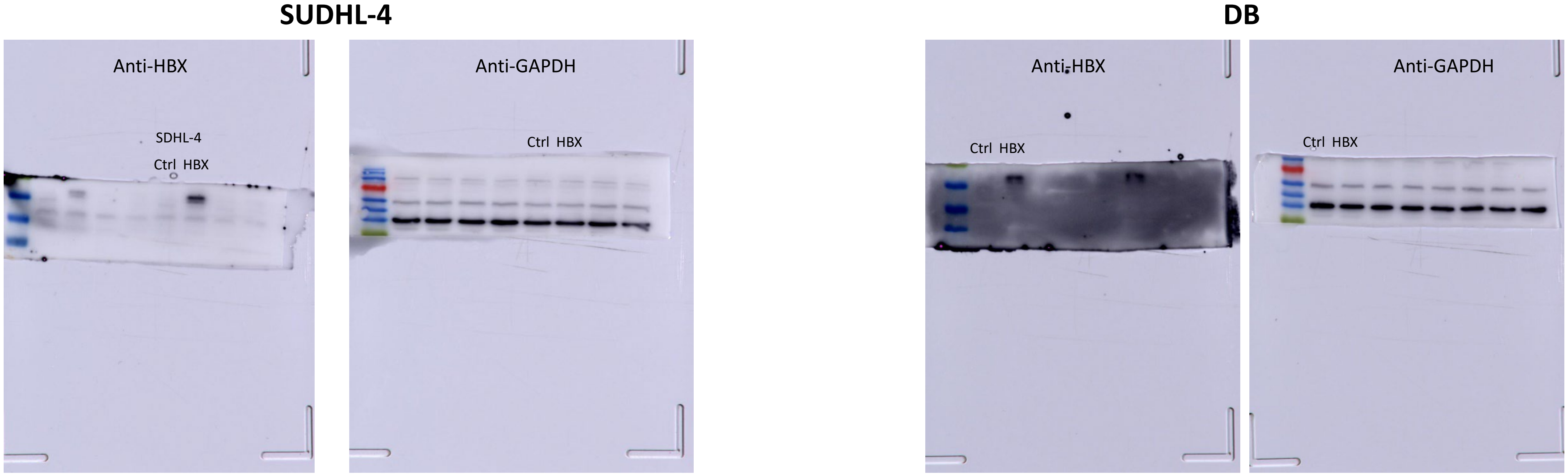
SUDHL-4
DB
Anti-HBX
SDHL-4
Ctrl HBX
Anti-GAPDH
Ctrl HBX
Anti-HBX
Ctrl HBX
Anti-GAPDH
Ctrl HBX

Figure3G

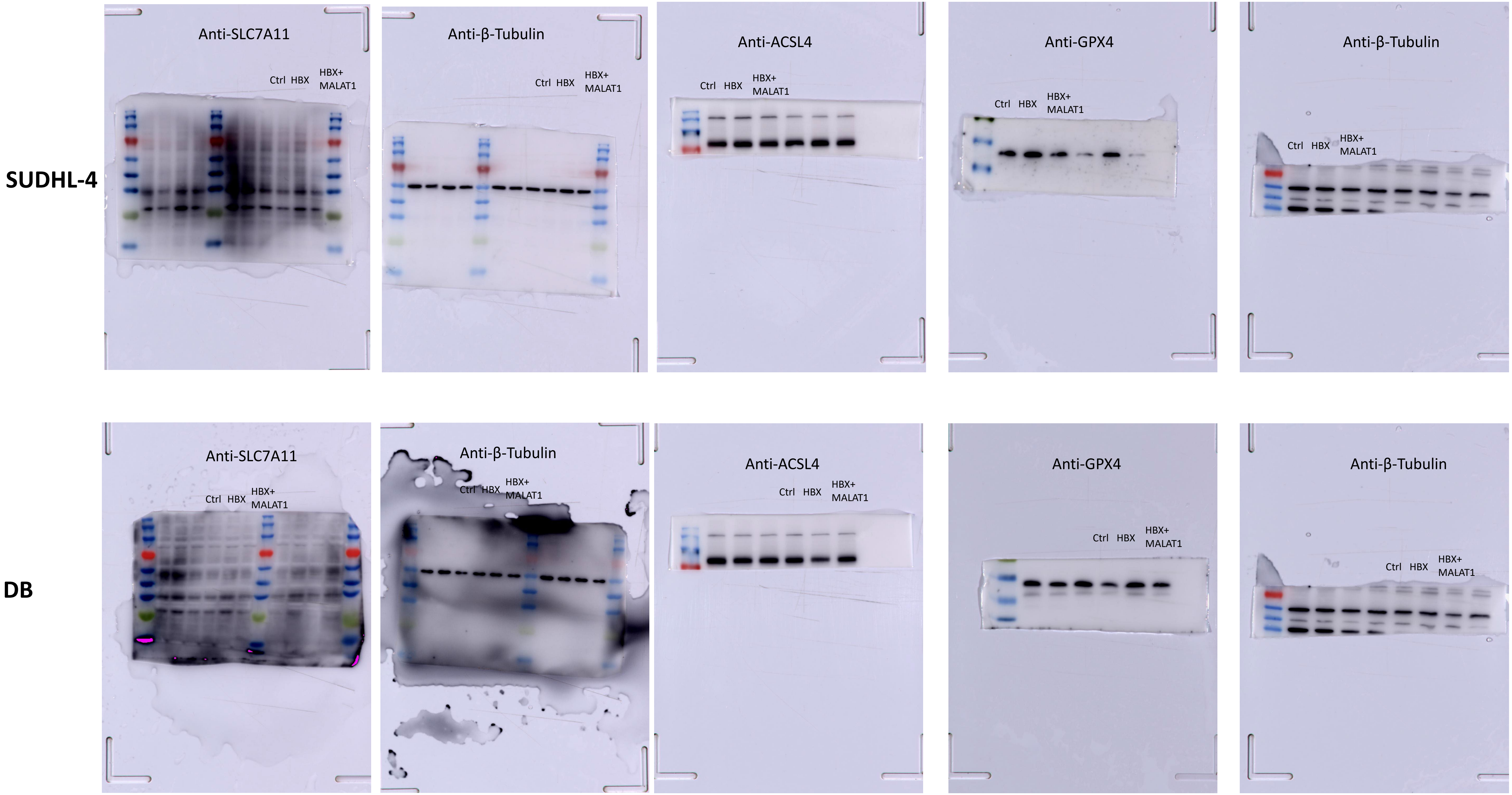
SUDHL-4
Anti-SLC7A11
Ctrl HBX HBX+ MALAT1
Anti-β-Tubulin
Ctrl HBX HBX+ MALAT1
Anti-ACSL4
Ctrl HBX HBX+ MALAT1
Anti-GPX4
Ctrl HBX HBX+ MALAT1
Anti-β-Tubulin
Ctrl HBX HBX+ MALAT1
DB
Anti-SLC7A11
Ctrl HBX HBX+ MALAT1
Anti-β-Tubulin
Ctrl HBX HBX+ MALAT1
Anti-ACSL4
Ctrl HBX HBX+ MALAT1
Anti-GPX4
Ctrl HBX HBX+ MALAT1
Anti-β-Tubulin
Ctrl HBX HBX+ MALAT1

Figure5J

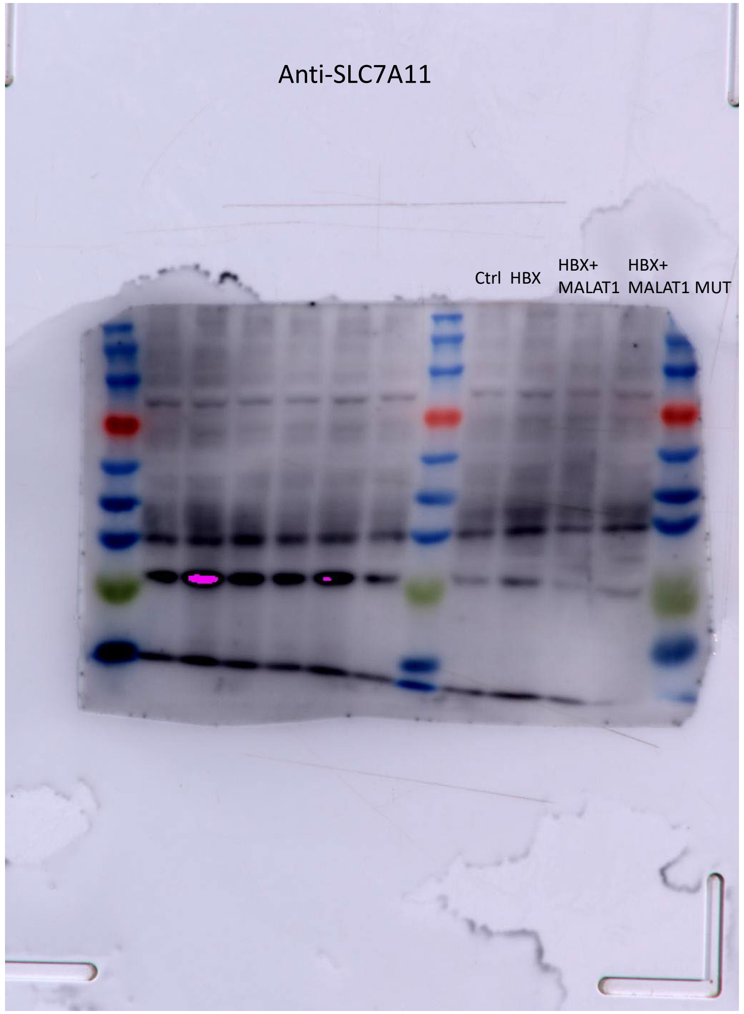
Anti-SLC7A11
Ctrl
HBX
HBX+ MALAT1
HBX+ MALAT1 MUT

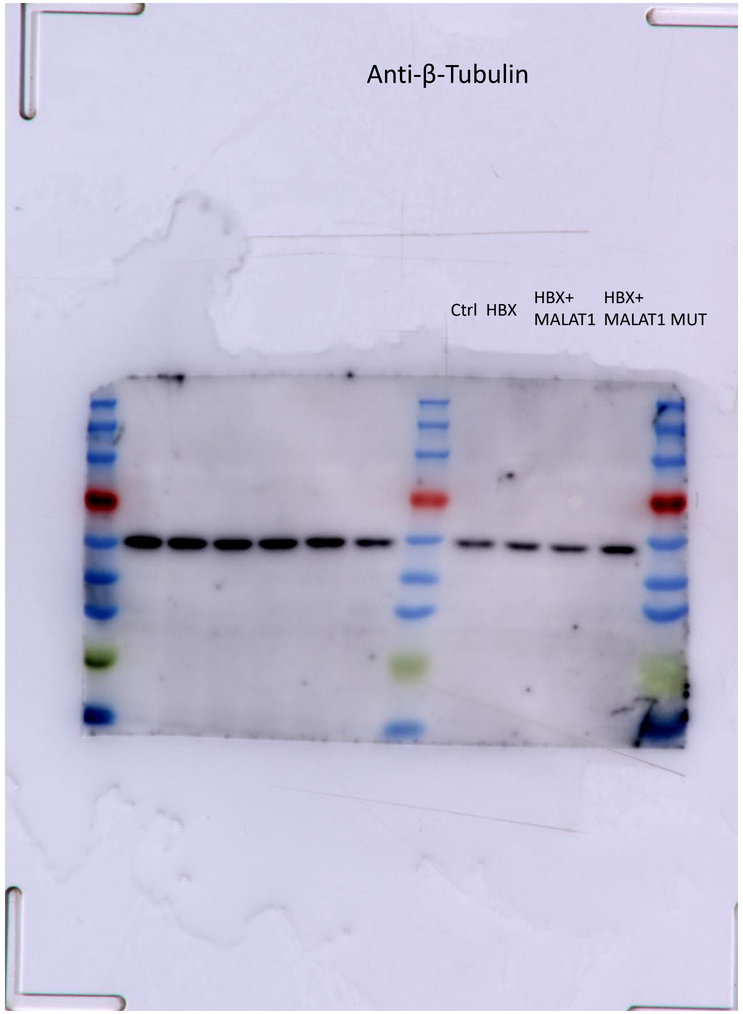
Anti-β-Tubulin
Ctrl
HBX
HBX+ MALAT1
HBX+ MALAT1 MUT

FigureS4

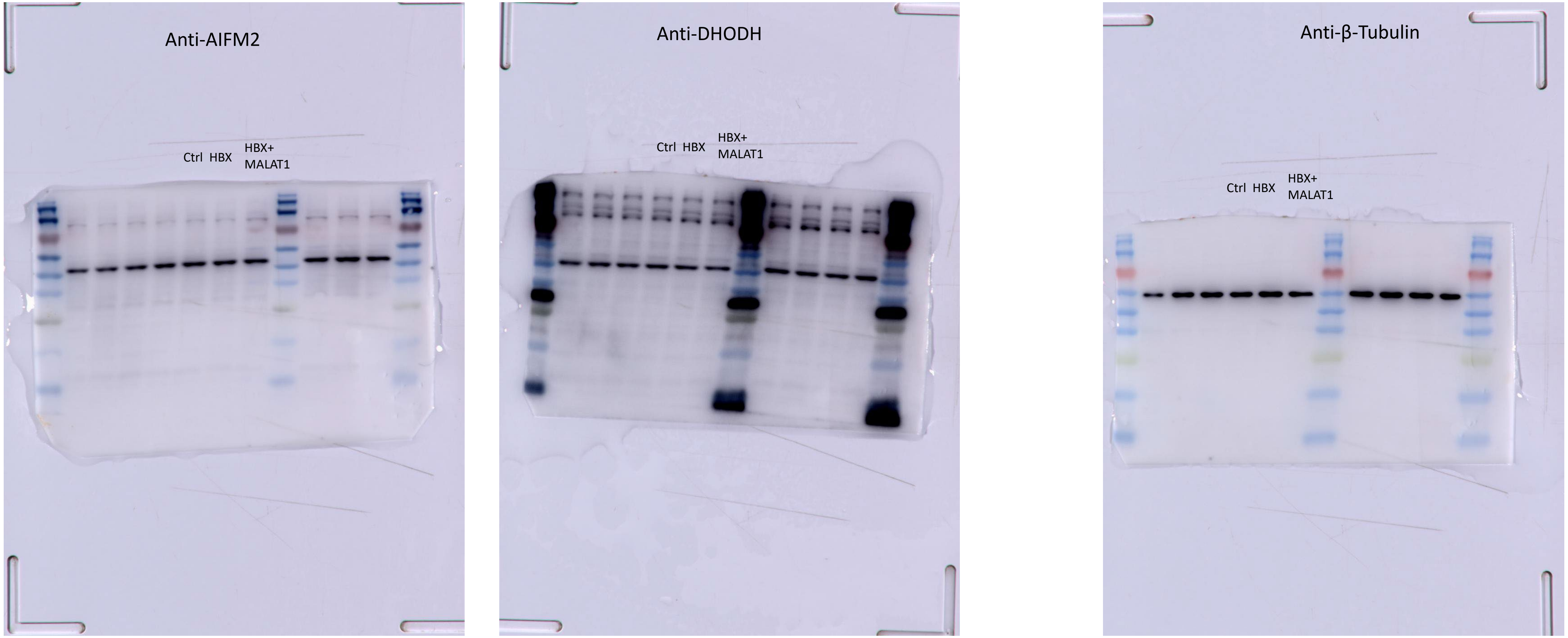
Anti-AIFM2
Ctrl HBX HBX+ MALAT1
Anti-DHODH
Ctrl HBX HBX+ MALAT1
Anti-β-Tubulin
Ctrl HBX HBX+ MALAT1

FigureS5C

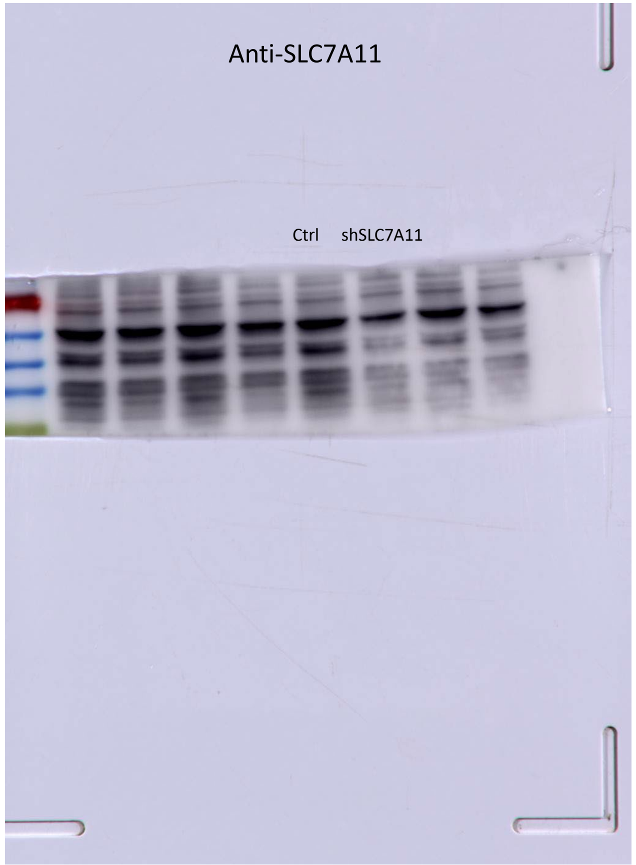

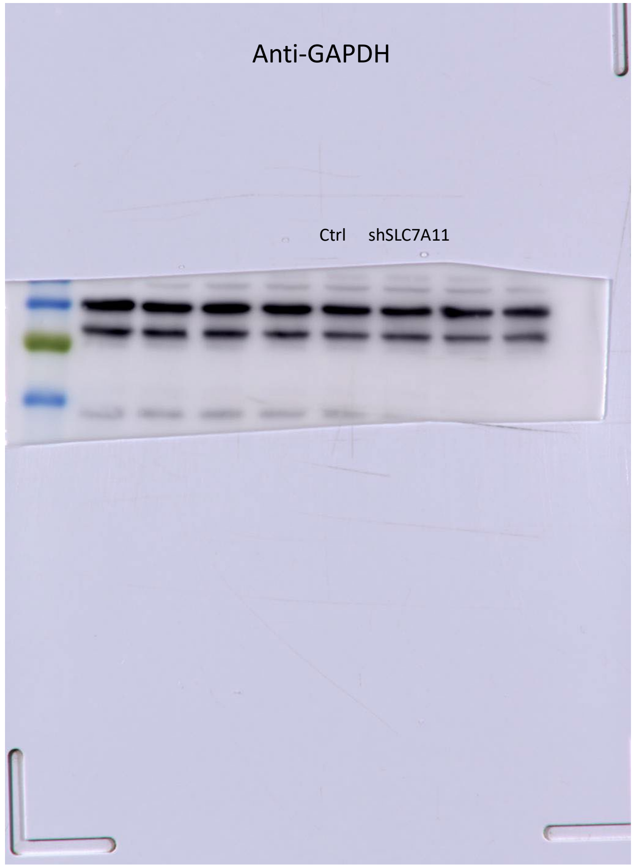

FigureS5D

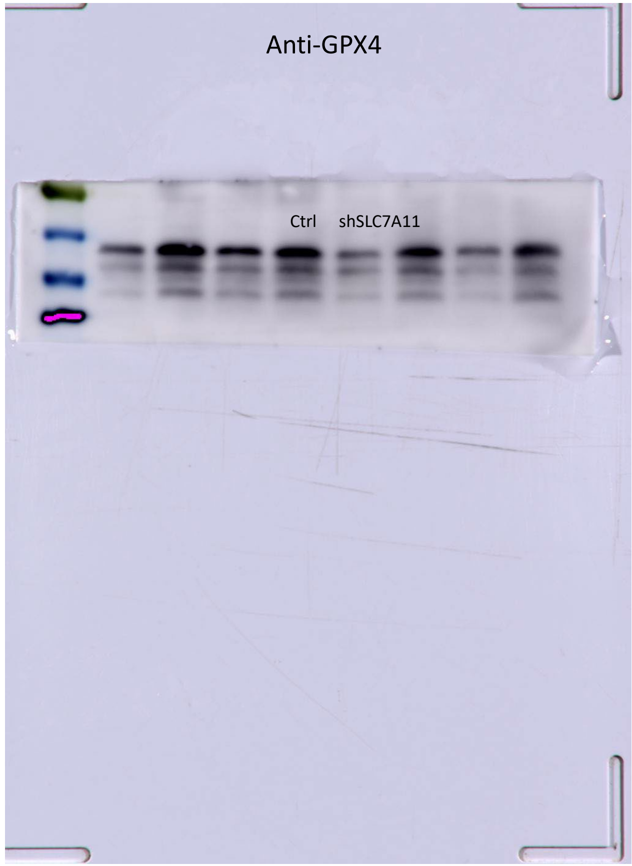

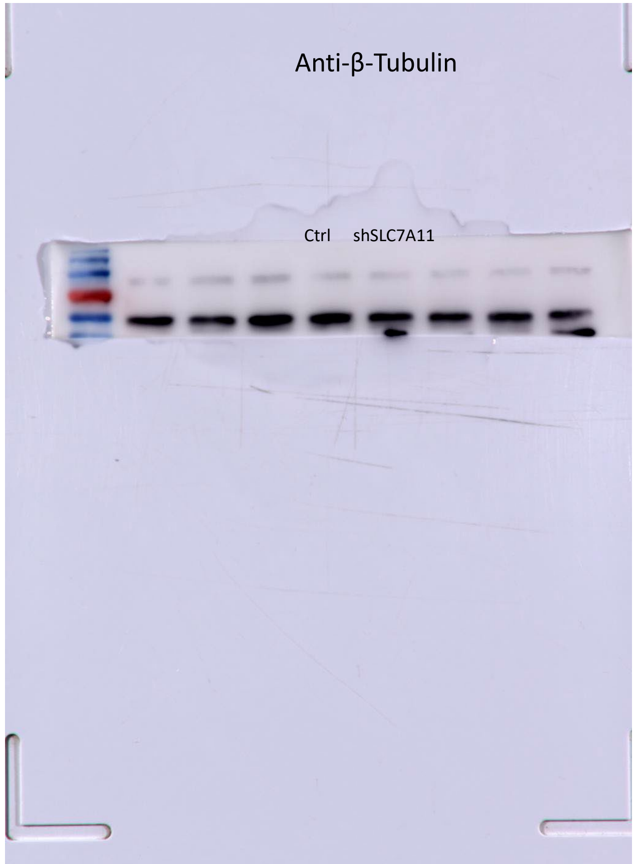

FigureS7C

Anti-SLC7A11 Anti-ACSL4 Anti-GPX4 Anti-β-Tubulin

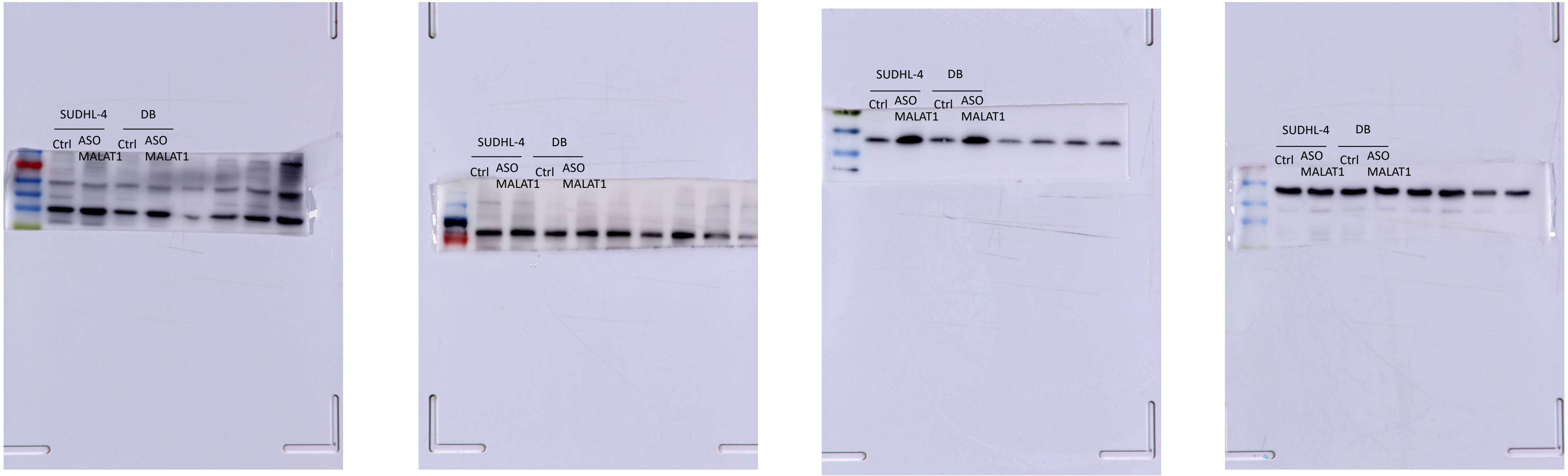

FigureS10D

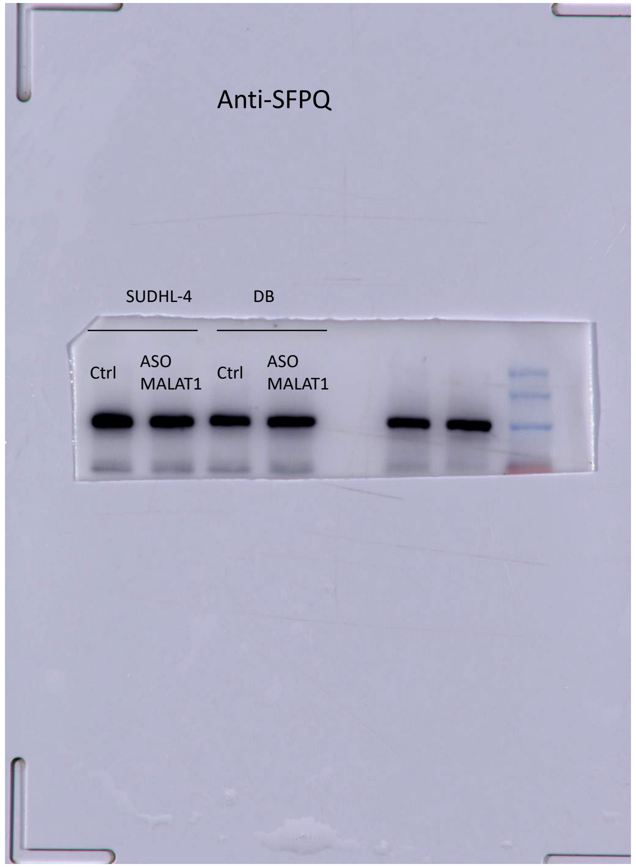
Anti-SFPQ
SUDHL-4
DB
Ctrl
ASO MALAT1
Ctrl
ASO MALAT1

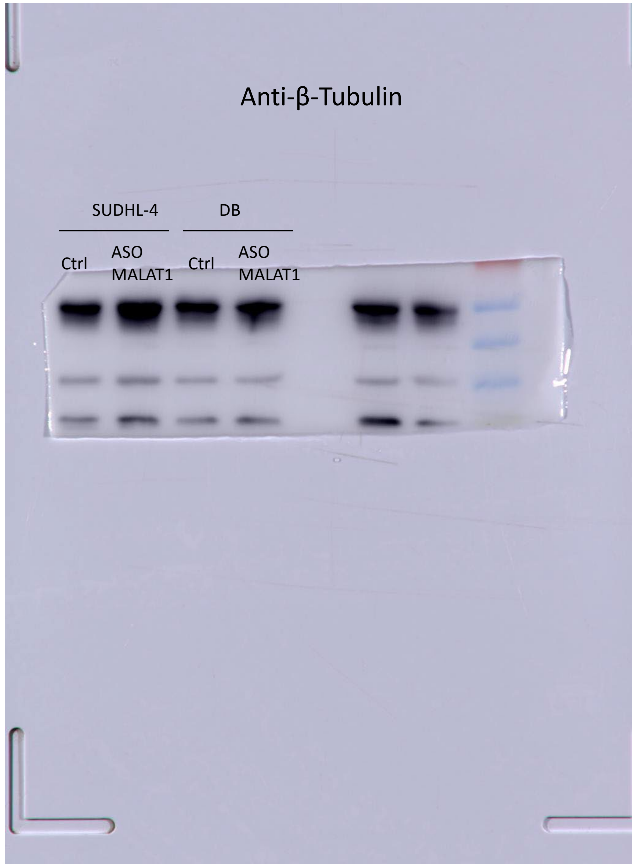
Anti-β-Tubulin
SUDHL-4
DB
Ctrl
ASO MALAT1
Ctrl
ASO MALAT1

FigureS10F

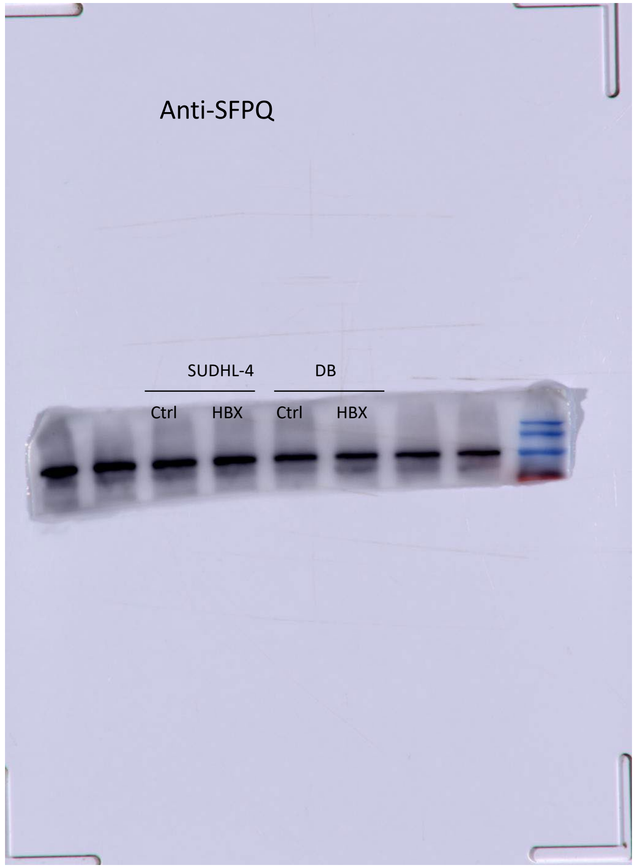
Anti-SFPQ
SUDHL-4
DB
Ctrl
HBX
Ctrl
HBX

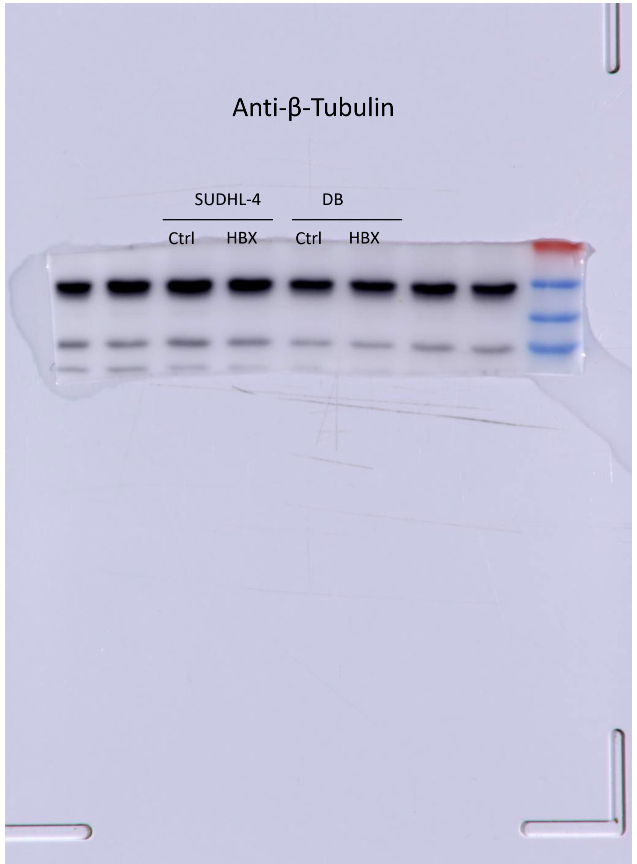
Anti-β-Tubulin
SUDHL-4
DB
Ctrl
HBX
Ctrl
HBX

FigureS12

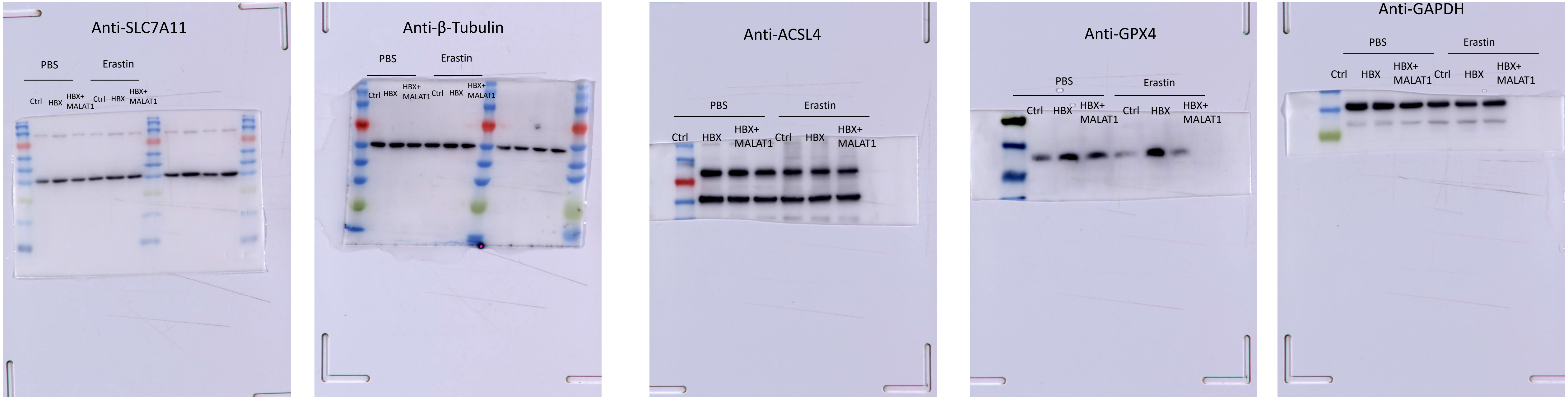
Anti-SLC7A11
PBS
Erastin
Ctrl
HBX
HBX+ MALAT1
Ctrl
HBX
HBX+ MALAT1
Anti-β-Tubulin
PBS
Erastin
Ctrl
HBX
HBX+ MALAT1
Ctrl
HBX
HBX+ MALAT1
Anti-ACSL4
PBS
Erastin
Ctrl
HBX
HBX+ MALAT1
Ctrl
HBX
HBX+ MALAT1
Anti-GPX4
PBS
Erastin
Ctrl
HBX
HBX+ MALAT1
Ctrl
HBX
HBX+ MALAT1
Anti-GAPDH
PBS
Erastin
Ctrl
HBX
HBX+ MALAT1
Ctrl
HBX
HBX+ MALAT1
